# Establishment of tumor inflammasome clusters with distinct immunogenomic landscape aids immunotherapy

**DOI:** 10.7150/thno.63202

**Published:** 2021-10-17

**Authors:** Qingyu Liang, Jianqi Wu, Xin Zhao, Shuai Shen, Chen Zhu, Tianqi Liu, Xiao Cui, Ling Chen, Chunmi Wei, Peng Cheng, Wen Cheng, Anhua Wu

**Affiliations:** 1 Department of Neurosurgery, The First Hospital of China Medical University, Shenyang, China.; 2 Department of Basic Medicine, China Medical University, Shenyang, China.; 3 Department of Neurosurgery, Chinese People's Liberation Army of China (PLA) General Hospital, Medical School of Chinese PLA, Institute of Neurosurgery of Chinese PLA, China.; 4 Department of Radiotherapy, The Affiliated Tumor Hospital of Guangxi Medical University, Nanning, China.

**Keywords:** Inflammasome, tumor classification, prognosis, tumor-associated macrophages, immunotherapy

## Abstract

Inflammasome signaling is a reaction cascade that influences immune response and cell death. Although the inflammasomes participate in tumorigenesis, their role as an oncogenic booster or a tumor suppresser is still controversial. Therefore, it is important to comprehensively investigate the inflammasome signaling status across various cancers to clarify its clinical and therapeutic significance.

**Methods:** A total of 9881 patients across 33 tumor types from The Cancer Genome Atlas database were included in this study. Five gene sets were identified to step-wisely profile inflammasome signaling. Unsupervised clustering was used for sample classification based on gene set enrichment. Machine learning and *in vitro* and *in vivo* experiments were used to confirm the implications of inflammasome classification.

**Results:** A hundred and forty-one inflammasome-signaling-related genes were identified to construct five gene sets representing the sensing, activation, and termination steps of the inflammasome signaling. Six inflammasome clusters were robustly established with distinct molecular, biological, clinical, and therapeutic features. Importantly, clusters with inflammasome signaling activation were found to be immunosuppressive and resistant to ICB treatment. Inflammasome inhibition reverted the therapeutic failure of ICB in inflammasome-activated tumors. Moreover, based on the proposed classification and therapeutic implications, an open website was established to provide tumor patients with comprehensive information on inflammasome signaling.

**Conclusions:** Our study conducted a systematical investigation on inflammasome signaling in various tumor types. These findings highlight the importance of inflammasome evaluation in tumor classification and provide a foundation for improving relevant therapeutic regimens.

## Introduction

Inflammasomes refer to the cytoplasmic multimeric protein complexes that sense pathogen- or danger-associated molecular patterns (PAMPs or DAMPs, respectively) to mediate the inflammatory response and induce programmed cell death known as pyroptosis 1. In the sensing step, PAMPs and DAMPs are first recognized by the inflammasome and form the inflammasome complex with pro-caspase-1 2. Then, in the activation step, the inflammasome complex (IC) activates caspase-1 that cleaves Gasdermin D (GSDMD) and proinflammatory cytokines IL-1β and IL-18. In the termination step, the cleaved GSDMD perforates the cytomembrane leading to the rapid release of activated IL-1β and IL-18 and cell pyroptosis 2. Therefore, the inflammasome signaling is a reaction cascade with multiple steps that affects the modulation of local inflammation and determines the cell fate. Systematic profiling of each step of the inflammasome signaling is essential to clarify its role in the pathology of diseases.

In recent years, increasing efforts have been made to clarify the role of inflammasome signaling in tumorigenesis. When activated, inflammasome signaling acts as a tumor promotor to amplify the undesirable chronic inflammatory response 3. Downstream effectors of inflammasome signaling, IL-1β, and IL-18, have also been demonstrated to promote tumor angiogenesis 4, metastasis 5, and immune evasion 6 through paracrine and autocrine mechanisms. Conversely, the tumor-suppressive function of inflammasome signaling has been recognized in colitis-associated cancer 7, 8. Therefore, the role of inflammasome signaling in tumors remains controversial and elucidating the implications of inflammasome signaling across different types of cancer is helpful for clarifying its role.

The immune checkpoint blockade (ICB) has been recognized as a promising approach for tumor treatment. However, owing to the intrinsic and extrinsic mechanisms, numerous solid tumors rapidly develop resistance against ICB 9. One of the most important factors contributing to this dilemma is the tumor immunosuppressive microenvironment, in which infiltrating immunosuppressive cells, such as tumor-associated macrophages (TAMs) and regulatory T cells (Tregs) 10, can induce CD8^+^ T-cell exhaustion by secreting immunosuppressive factors 11, 12. Recent studies obtained confusing observations that both activators 13, 14 and inhibitors 10 of inflammasome signaling can remold the tumor immunosuppressive environment, thus affecting the therapeutic response to the ICB treatment. Therefore, modulating the inflammasome signaling could be a promising approach to overcome ICB resistance 15, 16. However, the complex relationship among inflammasome status, tumor immunosuppressive microenvironment, and ICB resistance needs further investigation.

In this study, to systematically explore the role of inflammasome signaling in tumor biology and management, we profiled the reaction steps of inflammasome signaling based on five inflammasome-signaling-related gene sets across 33 tumor types. Six inflammasome clusters were summarized with a distinct genomic pattern, biological phenotype, and ICB treatment response. The Cancer Analysis of Inflammasome Balance (CAIB) website was established to provide guidance for tumor classification and treatment clinically.

## Methods

### Curation of inflammasome-signaling-related gene sets

**Figure 1:**
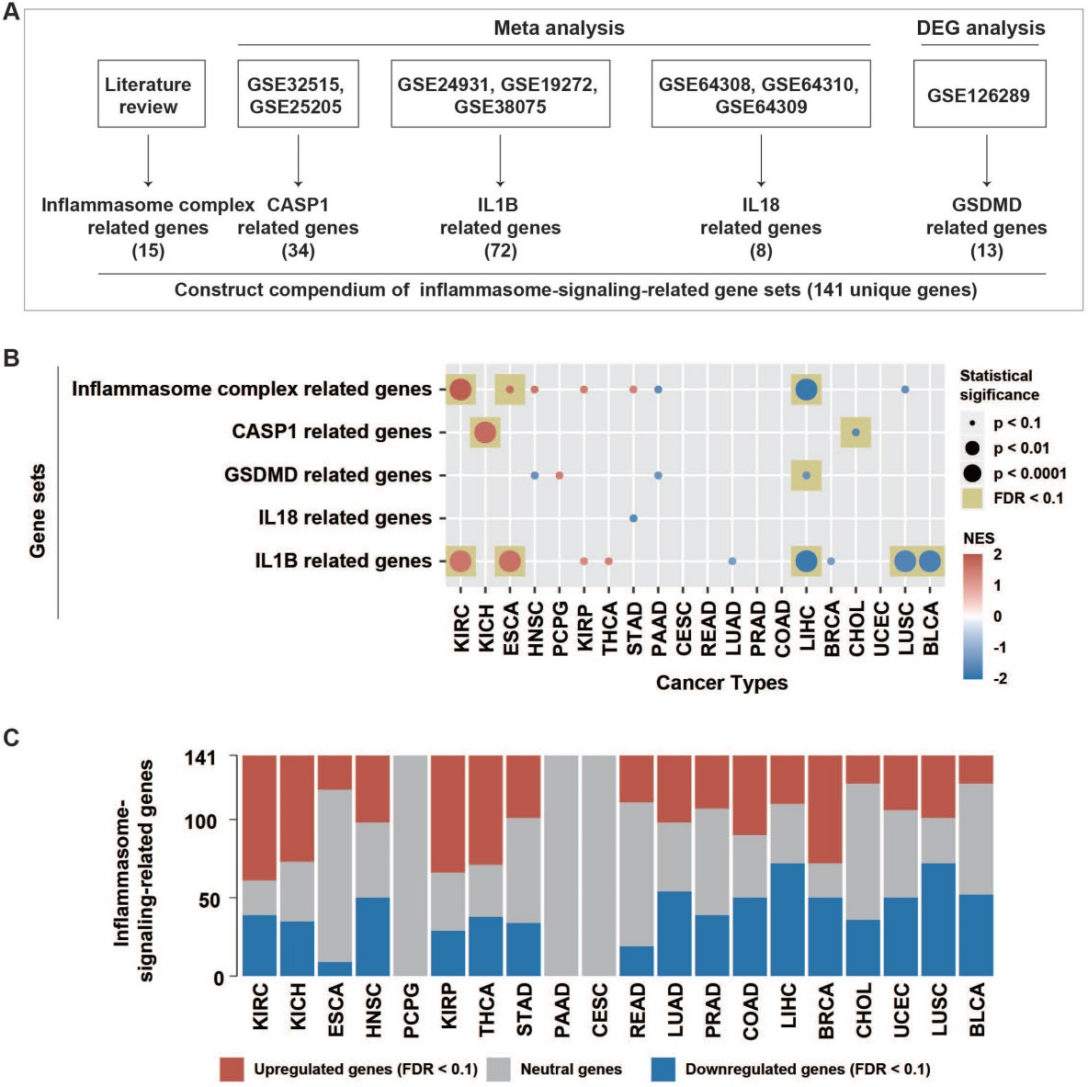
** Patterns of inflammasome signaling steps. (A)** Constructed compendium of inflammasome-signaling-related gene sets. **(B)** Enrichment or depletion of inflammasome-signaling-related gene sets was evaluated using GSEA in 20 tumor types with paired tumor and normal samples (more than two pairs). Colors in the circle represented the NES value. The size of the circle represents the p-value. Results with p-values less than 0.1 are shown. **(C)** Fractions of differentially expressed inflammasome-signaling-related genes in the 20 tumor types (Wilcoxon rank-sum test, FDR < 0.1). NES, normalized enrichment score; FDR, false discovery rate; DEG: differential expression gene.

Inflammasome signaling was mainly controlled by three sequential steps, including sensing (activated by inflammasome complexes), activation (activated by caspase-1), and termination (activated by GSDMD, IL1B, and IL18) (Figure 1A). In this study, we attempted to evaluate the inflammasome signaling steps based on five gene sets. Curated 15 genes correlating with inflammasome complexes (ICs) were collected from literature review 17. Because caspase-1 (CASP1), GSDMD, IL1B, and IL18 usually undergo extensive post-translational regulations as described previously 18, the expression of these genes may not accurately represent their activity. In a previous study, 34 CASP1-regulated genes and 72 IL1B-regulated genes were identified using a meta-analysis from the GEO dataset (Figure 1A, Table S1) 17. Here, we conducted a similar meta-analysis procedure based on three GEO datasets (GSE64308, GSE64309, and GSE64310). Then curated eight IL18-regulated genes were identified (Table S1).

Because only one GEO dataset (GSE126289), including two expression profiles, was derived after GSDMD manipulation, the meta-analysis failed to identify GSDMD-regulated genes. Therefore, we conducted differential expression analysis using limma R package on these two expression profiles, respectively. Finally, 13 GSDMD-regulated genes were identified by screening out differential expression genes (DEGs) from the two profiles (|logFC| >1, p-value < 0.05) (Table S1).

### Datasets collection and processing

#### Mutation data

TCGA pan-cancer somatic data (mc3.v0.2.8.PUBLIC.xena) was obtained from the University of California, Santa Cruz Xena (UCSC Xena; https://xenabrowser.net/datapages/). The related maf file (mc3.v0.2.8.PUBLIC.maf) was retrieved from syn7824274 on the Synapse website (https://www.synapse.org). Mutation fraction only derived from non-silent somatic mutations. Two hundred ninety-one high-confidence driver genes were retrieved from Tamborero's study 19. A chi-square test was used to evaluate the differential distribution of non-silent mutations of driver genes between one cluster and all other clusters.

#### Somatic copy-number alteration (SCNA) data

Pan-cancer level gistic2 data of TCGA were derived from UCSC Xena, which were used to explore the distribution of SCNA across clusters at the pan-cancer level. Eighty-four driver focal locus of SCNA were collected from Hoadley's study 20. A chi-square test on the frequency within one cluster compared to all other clusters was conducted for driver focal locus.

#### DNA methylation data

TCGA pan-cancer DNA methylation 450k data was obtained from UCSC Xena. According to the annotation file for methylated probes, Probes with distance to transcription start site (TSS) ≤ 1500 bases were selected for further analysis. When a gene correlated with multiple TSS1500 probes, only the probe with largest maximum absolute deviation (MAD) was selected. To explore the potential mechanisms underlying dysregulated IRGs, Pearson analysis was performed between methylated level and expression level of each gene, where the significant correlation was determined with a false discovery rate (FDR) less than 0.001. Driver TSS1500 probes were selected mainly based on the criteria described in Hoadley's study 21. Briefly, TSS1500 probes with mean β-value < 0.2 and β-value > 0.3 in no more than five samples across pan-normal tissue and β-value ≥ 0.3 in more than 10% of tumors across 33 tumor types were selected for further filtering in the champ.filter function of ChAMP R package. Finally, 6085 driver TSS1500 probes were identified for differential methylation analysis between one cluster and all other clusters using the champ.DMP function. Probes with absolute logFC > 0.2 and FDR < 0.01 were considered to be significantly dysregulated.

#### RNA data

TCGA RNA-seq data for 33 tumor types and CCLE RNA-seq data were acquired from the Google Cloud Pilot RNA-sequencing for CCLE and TCGA project (https://osf.io/gqrz9/), which were upper-quartile normalized and log-transformed as described in a previous study 22. CGGA GBM RNA-seq data with clinical information was downloaded from the CGGA mRNAseq_325 dataset (http://www.cgga.org.cn). TCGA clinical information was collected from UCSC Xena. We used the IMvigor R package to retrieve RNA-seq counts data (IMvigor210) with detailed clinical information from patients after anti-PD-L1 treatment, which was also processed using the same method as in CCLE and TCGA data 11. RNA data of cutaneous melanoma samples collected before anti-PD1 therapy were retrieved from three cohorts (dbGaP: phs001036, GSE91061, and GSE78220). RNA data of colon cancer with immunohistochemistry (IHC) information of macrophages was retrieved from GSE39582. Pan-cancer miRNA microarray data of TCGA were also downloaded from UCSC Xena. The sample number of each dataset is described in Table S2. Protein-coding RNA and long non-coding RNA (lncRNA) were annotated based on the Ensembl GRCh38.84 version (http://www.ensembl.org/Homo_sapiens/Info/Index). Differential expression analysis of tumor vs. their matched normal samples in 20 tumor types with more than two tumor-normal pairs was evaluated by the Wilcoxon rank-sum test. For comparing one cluster to other clusters, the limma package (voom with quantile normalization just for RNA-seq counts data) was used to perform differential expression on coding RNA, lncRNA, and miRNA. Coding RNA and miRNA with absolute logFC > 1 and FDR < 0.01 and lncRNA with absolute logFC > 2 and FDR < 0.01 were considered to be differentially expressed.

#### Reverse Phase Protein Array (RPPA) and drug-sensitive data

RPPA data was collected from UCSC xena, while drug-sensitive data on cell lines matched with CCLE was retrieved from Genomics of Drug Sensitivity in Cancer (GDSC, https://www.cancerrxgene.org/). The differential analysis in RPPA and drug-sensitive (IC50) data was performed using the Wilcoxon rank-sum test.

### Calculation of scores on gene sets and biological features

ssGSEA was used to calculate score of gene sets, including the established five inflammasome-signaling-related gene sets, immunosuppressive gene sets, immune-cell-related gene sets, and gene programs and pathway signature 23-26. Among the five inflammasome-signaling related scores, CASP1, GSDMD, IL1B, and IL18 scores were generated by subtracting the negative-regulated score from the corresponding positive-regulated score. Tumor purity is defined as the fraction of tumor cell content in the tissue 27. The stromal score, immune score, and tumor purity were calculated using the ESTIMATE R package 28. Number of segments and Aneuploidy were retrieved from complementary tables in Thorsson's study 29.

### Inflammasome subtypes clustering and prediction

The matrix of five inflammasome-signaling-related scores was used as the input data matrix in the Consensus Cluster Plus R package to determine the optimal number of inflammasome clusters for 9881 tumor samples. Euclidean distance and K-means clustering were used. Cluster-consensus and delta area from unsupervised consensus clustering and average silhouette width calculated from the Silhouette R package were used to confirm the stability of the clustering. To predict inflammasome clusters in external datasets based on the five scores, a two-layer validation strategy was used to compare the prediction accuracies of six machine learning algorithms including Classification and Regression Trees (CART), Logistic Regression (LR), Linear Discriminant Analysis (LDA), KNeighbors Classifier (KNN), Gaussian NB (NB), and Support Vector Machine (SVM) in TCGA pan-cancer data set. Briefly, TCGA samples were split randomly into training (80%) and validation (20%) sets. The training set was then used to compare the prediction accuracies of six algorithms by five-fold cross-validation process to overcome overfitting. The accuracies of six algorithms were further assessed using the validation set as an external-layer evaluation. Then, SVM with the highest prediction accuracy (96%; Table S3) was applied to external datasets (CCLE, CGGA GBM, IMvigor210, dbGaP: phs000452, GSE78220, and GSE91061).

### Tumor map analysis

We used dist function in R to calculate the Euclidean distance from the matrix of five inflammasome-signaling-related scores across TCGA 33 tumor types. Euclidean distance was used to calculate Euclidean similarity by the formula Euclidean similarity = (1/(1 + Euclidean_distance)), as described in previous study 21. Finally, the matrix of Euclidean similarity was used as input to generate a tumor map on the TumorMap website (https://tumormap.ucsc.edu/).

### Functional enrichment analysis using GSEA

For GSEA analysis performed in clusterProfiler R package, the pre-ranked gene lists, based on signed negative log_10_FDR from the Wilcoxon rank-sum test on the comparison between tumors and paired normal tissues, were run against the inflammasome complex related, CASP1 related, GSDMD related, IL18 related, and IL1B related genes, while the pre-ranked gene lists, based on logFC from limma analysis on comparing one cluster to all other clusters, were run against pathways from Kyoto Encyclopedia of Genes and Genomes (KEGG), REACTOME, and BioCarta pathways downloaded from MSigDB.

### Cell lines and cell culture

GBM cell lines GL261, U87MG, T98, and BRCA cell lines MB231 (MDA-MB-231), MB468 (MDA-MB-468) were purchased from the American Type Culture Collection (ATCC, Manassas, VA, USA). THP-1 was purchased from National Collection of Authenticated Cell Cultures (NCACC, Shanghai, China). SB (Sleeping Beauty) mGSC (mouse glioma sphere cell) was harvested from *de novo* induced spontaneous GBM model as previously described 30. B16F10 was purchased from Procell Life Science & Technology (Hyderabad, Telangana, India). GBM cell lines were maintained in Dulbecco's modified Eagle's medium (DMEM) (Biological Industries, Beit HaEmek, Israel), BRCA cell lines were maintained in Leibovitz L15 medium (Gibco, USA), THP-1 and B16F10 were maintained in RPMI 1640 medium (RPMI) (Biological Industries). All culture mediums were supplemented with 10% heat-inactivated fetal bovine serum (FBS) (Gibco) and 1:1000 Penicillin-Streptomycin (Gibco). SB mGSC was cultured in stem cell medium (Neurobasal-A medium with B27 supplement, 10 ng/mL EGF, and 10 ng/mL FGF). GBM, THP-1, and B16F10 cell lines were cultured in humidified atmosphere at 37 °C in 5% CO_2_, whereas BRCA cell lines were cultured in humidified atmosphere at 37 °C in 0% CO_2_.

### CASP1 knockdown

siRNAs targeting human CASP1 was designed and constructed in Sangon Biotech (Shanghai, China). siRNA1: sense (5′-3′): CACACGUCUUGCUCUCAUUAUTT, antisense (5′-3′): AUAAUGAGAGCAAGACGUGUGTT; siRNA2: sense (5′-3′): GAAGAGUUUGAGGAUGAUGCUTT, antisense (5′-3′): AGCAUCAUCCUCAAACUCUUCTT. Lipofectamine™ 3000 Reagent (Invitrogen, Waltham, MA, USA) was used to perform siRNA transfection in cancer cells. Cells were then incubated for 48 h before the next experiment.

### ELISA

Human and mouse IL-1β detecting ELISA kits were purchased from R&D System (Minneapolis, MN, USA). Cancer cells were incubated with Methylene Blue (MB) (MedChemExpress, HY-14536) or siRNAs or THP-1 for 48 h. Next, the complete cultured medium was replaced by a culture medium without FBS. After 24 h culture, the conditioned medium was collected for ELISA analysis. ELISA was performed following the manufacture's instruction. The optical density of each well was determined immediately using the microplate reader (VICTOR Nivo^TM^, Waltham, MA, USA) set to 450 nm.

### TAM migration assay

TAM was induced by co-culturing THP-1 with cancer cells for 48 h. TAM from the co-culturing systems was plated into 5.0 μm 24-well transwell inserts at a concentration of 5 × 10^4^ cells per well in 200 μl serum free RPMI. 600 μl of serum free medium was added to the under receiver well where MB or siRNA pre-treated cancer cells were plated. TAM was allowed to migrate downward for 24 h. The non-migrated cells on the upper chamber were removed using a cotton swab, and the migrated cells were fixed and stained with 1% crystal violet. Stained cells were photographed using a microscope (Olympus, Tokyo, Japan) and quantified using the ImageJ software (National Institutes of Health, Bethesda, MD, USA).

### Western blotting analysis

Cultured cells or minced tumor tissue were harvested at indicated times and lysed using the Cell lysis buffer (P0013J, Beyotime) containing 1% PMSF (ST506, Beyotime, China) at 4 °C for 30 min. Proteins were heat denatured at 100 °C for 10 min before separating by electrophoresis in SDS-PAGE (P0012A, Beyotime) and transferred to PVDF membrane (FFP28, Beyotime). After blocking with 5% skim milk for 2 h, the PVDF membranes were incubated with primary antibodies of hPD-L1 (Abcam, Cambridge, UK, ab213524), mPD-L1 (Abcam, ab213480), hNlrp3 (Abcam, ab214185), mNlrp3 (Abcam, ab270449), hAim2 (Abcam, ab93015), mAim2 (Proteintech, 66902-1-Ig), GAPDH (PTG, 60004-1), hCleaved Caspase 1 (CST, D57A2), mCleaved Caspase-1 (Asp296) (E2G2I) (CST, #89332) overnight at 4 °C. The membranes were then incubated with the HRP-conjugated secondary antibodies (SA00001-1 and SA00001-2, Proteintech, Rosemount, IL, USA) for 1 h, and visualized using Western Blotting Luminol Reagent (sc-2048, Santa Cruz Biotechnology, CA, USA).

### RT-qPCR

Total RNA was extracted from cancer cells or TAM from the co-culturing systems using TRIzol (TaKaRa, Kyoto, Japan) following the manufacturer's instruction. mRNA was reverse-transcribed to cDNA using PrimeScript™ RT Master Mix (RR036A, Takara, Shiga, Japan). Amplification reaction assays containing TB Green® Premix Ex Taq™ (RR420A, Takara) were detected by LightCyclerR480 (Roche Diagnostics Ltd., Basel, Switzerland) under identical amplification conditions. 18S was used as the reference gene for normalization, and mRNA abundance was quantified using the threshold cycle method. Each reaction was performed in triplicate. The primers used are listed in Table S12.

### Immunohistochemical (IHC) staining

Informed consent was obtained from all glioma patients, and the use of human samples for IHC was approved by the Institutional Review Board of The First Affiliated Hospital of China Medical University. After deparaffinization of the paraffin-embedded human GBM samples (n = 26) and cancer orthotopic/subcutaneous tumors, IHC staining was performed by Universal SAP Kit (universal Mouse/rabbit kit) (ZSGB-Bio, China, SAP-9100), and visualization was performed using DAB Color development kit (ZSGB-Bio, ZLI-9018) according to the manufacturer's instructions. Primary antibodies used were: NLRP3 (ab214185, Abcam), AIM2 (ab93015, Abcam), CD4 (#27520, CST), CD8 (ab217344, Abcam), Iba-1 (ab178846, Abcam), F4/80 (ab111101, Abcam), pro-Caspase3 (ab32499, Abcam), and Ki67 (ab15580, Abcam). Positive cell count was determined from three separate fields in each tumor sample. The semiquantitative evaluation of IHC staining was carried out using the immune score based on the percentage of stained cells and staining intensity as described 27.

### Flow cytometry analysis

Tumor masses were cut into pieces and digested into mononuclear cell suspension according to the protocol 31. Then, mononuclear cell suspension was blocked with anti- mouse CD16/32 (BD Biosciences, San Jose, CA, USA), followed by incubating at 4 °C for 30 min with fluorescein-conjugated specific antibodies against surface antigens CD45 (#563891, BD Pharmingen, CA, USA), CD11b (#553310, BD Pharmingen), F4/80 (#565411, BD Pharmingen), MHCII (#557000, BD Pharmingen), CD206 (FAB25351R-100UG, R&D Systems), CD3e (#551163, BD Pharmingen), CD4 (#552051, BD Pharmingen), and CD8a (#553030, BD Pharmingen). Then, intracellular staining was performed using Flow Cytometry Fixation & Permeabilization Buffer Kit I (#FC009, R&D Systems). IFN-γ (#554412, BD Pharmingen) and TNF-α (#554420, BD Pharmingen) antibodies were used for intracellular staining. Matched non-specific isotype immunoglobulins were stained as controls. 7-ADD attaining was used to exclude dead cells. After washing twice with staining buffer, cells were resuspended in 300 μl of PBS with 1% FBS and analyzed using a BD LSRFortessa flow cytometer (BD Biosciences). Results were processed and visualized with FlowJo V10 software (TreeStar, Ashland, OR, USA).

### Construction and treatment of the tumor models

GBM orthotopic model: SB mGSC (5 × 10^3^/mouse) or GL261 (10^5^/mouse) were stereotactically injected into the right striatum of six-to-eight-week-old male C57BL/6N mice (Charles River). Melanoma subcutaneous model: B16F10 (2 × 10^5^) was subcutaneously injected into the right axilla of six-to-eight-week-old male C57BL/6N mice (Charles River). Following the tumor injection, mice were randomly divided into four groups: control, anti-mouse PD-L1 mAb (Bio X Cell, BE0101) only, Methylene Blue or Belnacasan (MedChemExpress, HY-13205) only, and the combined treatment group. Methylene blue (3 mg/kg body weight) was intraperitoneally injected once every two days for two weeks beginning after 3^rd^ day of tumor injection. Belnacasan (50 mg/kg body weight) was intraperitoneally injected every day for two weeks beginning after 3^rd^ day of tumor injection. Anti-mouse PD-L1 mAb (200 µg/mice) was intraperitoneally injected once every three days for three times beginning after 5^th^ day or 7^th^ day of tumor injection in GBM model or melanoma model, respectively. For the melanoma model, tumor volume was measured every five days, and tumor volume was calculated according to the formula: length × (width)^2^ × 1/2. 20 days after the tumor injection, tumor-bearing mice were sacrificed, and tumor masses were resected for further analysis.

### Statistical analysis

Prism 7 v.7.0a and R v3.5.0 (http://www.R-project.org) software were mainly used for statistical analysis, unless stated otherwise. Differences between the two groups were assessed by Wilcoxon rank-sum test, Welch *t*-test, or chi-square test. The log-rank test was performed to estimate Kaplan-Meier survival curves. Prognostic factors were identified by univariate Cox regression analysis. A two-sided P-value < 0.05 was considered statistically significant unless otherwise stated. False positive rates were reduced by conducting Benjamini and Hochberg (BH or its alias “FDR”) correction in multiple tests.

## Results

### Patterns of inflammasome signaling steps

To stepwise explore the tumor inflammasome signaling, a total of 141 genes from five gene sets representing the sensing (15 IC-related genes), activation (34 CASP1-related genes), and termination (13 GSDMD-, 72 IL1B-, and eight IL18-related genes) steps of inflammasome signaling were identified. (Methods, Figure 1A, Figure S1, Table S1). The inflammasome signaling patterns were explored by comparing between tumor and paired normal samples in 20 tumor types. The five inflammasome-signaling-related gene sets were enriched in three types of tumors (KIRC: Kidney renal clear cell carcinoma, KICH: Kidney Chromophobe, and ESCA: Esophageal carcinoma), and more inflammasome-signaling-related genes were consistently upregulated in these tumors (Figure 1B-C, Figure S2, Table S4). However, four tumor types (LIHC: Liver hepatocellular carcinoma, CHOL: Cholangiocarcinoma, LUSC: Lung squamous cell carcinoma, and BLCA: Bladder Urothelial Carcinoma) showed a contrasting pattern. Additionally, we observed a similar pattern between IC- and IL1B- related genes in tumors (Figure 1B, Table S4). These findings suggest that there are distinct inflammasome status among tumor types.

### Inflammasome clusters with important clinical implications

**Figure 2:**
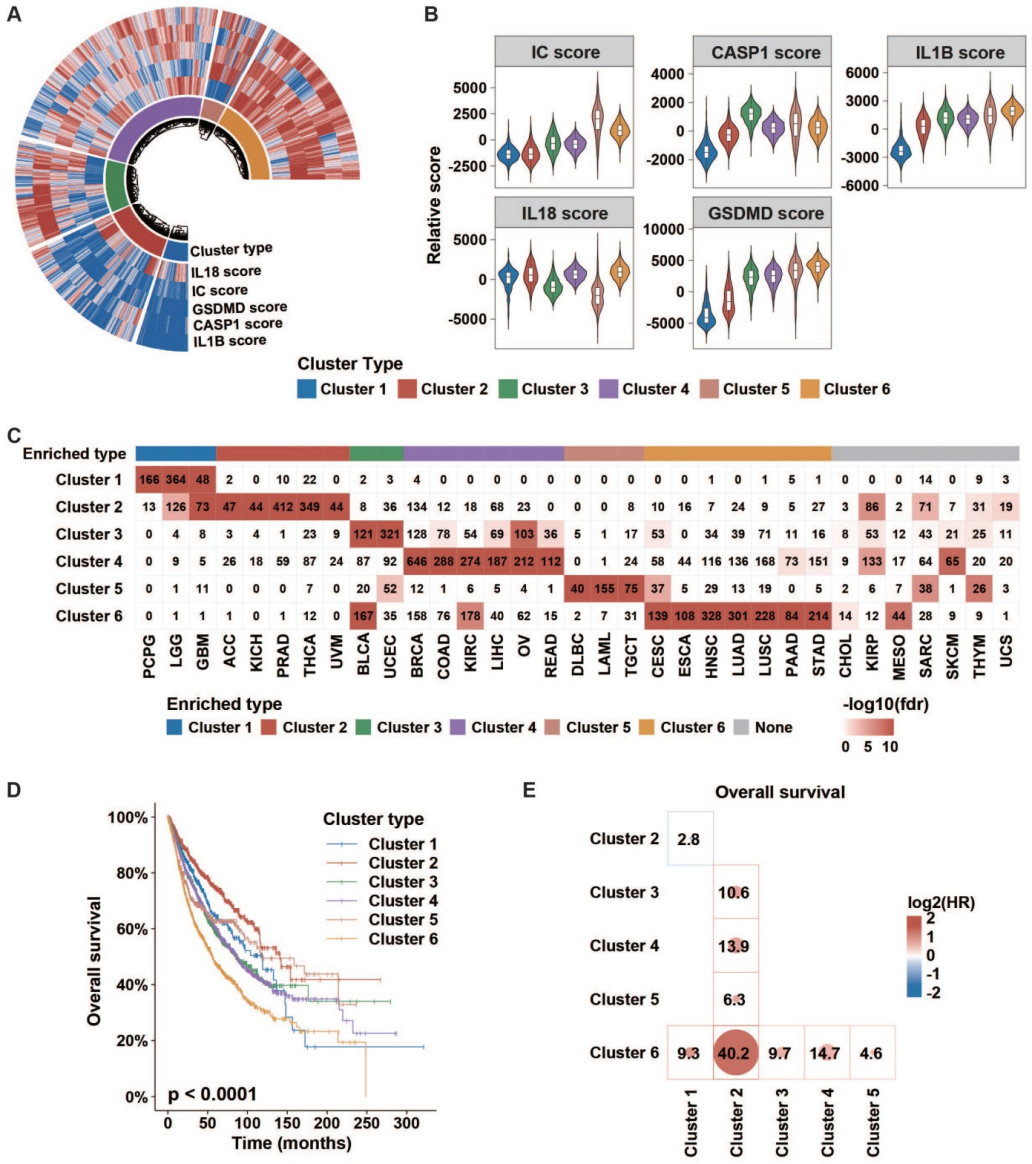
** Clinical importance of inflammasome clusters. (A and B)** Distribution of each inflammasome-signaling-related score in each cluster type. **(C)** Cases number in each inflammasome cluster across tumor types. Negative log10(FDR) is represented by colored boxes. The FDR was calculated in a hypergeometric test comparing the fraction of samples of a given tumor type in a cluster to the fraction of samples that are in that overall cluster. Increasing levels of enrichment were colored from white to red. Cases with enrichment threshold of more than 10 were grouped. **(D)** KM curve depicted the overall survival of cases from each inflammasome cluster. The p-value was evaluated using the log-rank test. **(E)** Cox analyses among clusters (cluster in row versus cluster in column) in whole samples were performed to evaluate the prognostic value. Colors in plot represent the HR value. The size of the circle and number represent the negative log10(p-value). Results with p values less than 0.05 are shown. IC, inflammasome complex; KM, Kaplan-Meier; HR, hazard ratios.

Then, we conducted an unsupervised consensus cluster analysis of 9881 samples across 33 tumor types based on the ssGSEA scores of five inflammasome-signaling-related gene sets. After evaluating the cluster consensus, delta area, and average silhouette width, six robust inflammasome clusters were established for all tumor samples (Figure 2A, Figure S3, Table S5A). As shown in Figure 2A-B and Table S6, clusters 1 and 2 were characterized by a low IC score with relatively low and high IL1B scores, respectively (IC^Low^IL1B^Low^ and IC^Low^IL1B^High^). Clusters 3 and 4 were characterized by a middle IC score with relatively high CASP1 and high IL18 scores, respectively (thereafter, IC^Mid^CASP1^High^ and IC^Mid^IL18^High^); and clusters 5 and cluster 6 were characterized by high IC score with relatively low IL18 and high IL18 scores, respectively (thereafter, IC^High^IL18^Low^ and IC^High^IL18^High^).

Next, we sought to explore the distribution of inflammasome clusters within each tumor type. Hypergeometric tests were used to evaluate the enrichment score. We found that PCPG, LGG, and GBM were over-enriched in cluster 1; ACC, KICH, PRAD, THCA, and UVM in cluster 2; BLCA and UCEC in cluster 3; BRCA, COAD, KIRC, LIHC, OV, and READ in cluster 4; DLBC, LAML, and TGCT in cluster 5; and CESC, ESCA, HNSC, LUAD, LUSC, PAAD, and STAD in cluster 6 (Figure 2C, Table S7). Seven tumor types showed relatively even distribution across six clusters. Additionally, we depicted the Sankey diagram to explore the correlation of TCGA tumor type, inflammasome cluster, and tissue system, which indicated that the distribution of inflammasome clusters was not affected by tumor histology (Figure S4A). In Ock's study, TCGA tumor was identified as the immunogenic/inflamed (TMIT I and IV) or cold (TMIT II and III) tumors according to the expression of PD-L1 and CD8A 32. The results of tumor microenvironment immune types (TMIT) from the Ock's study were retrieved to explore the distribution of inflammasome clusters further across immunogenic/inflamed and cold tumors. We found that immunogenic/inflamed tumors were more likely to be annotated as IC^Mid^ and IC^High^ tumors (Figure S4B), indicating a relationship between inflammasome clusters and TMIT subtyping system.

Next, we found that different clusters showed distinct survival prognosis (Figure 2D-E, Figure S5A-B). Tumors stratified into Cluster 6: IC^High^IL18^High^ correlated with worse prognosis. Furthermore, the relationship between inflammasome clusters and clinical outcome in KIRP and GBM showed a similar trend as that obtained by pan-cancer analysis, revealing that the high activity of inflammasome signaling indicates poor prognosis (Figure S5C). Taken together, the status of inflammasome signaling may have great influences on clinical prognosis.

### Genomic determinants of inflammasome clusters

To gain insights on the features of each inflammasome cluster, we sought to identify their multi-omics features.

#### Cluster 1

**Figure 3:**
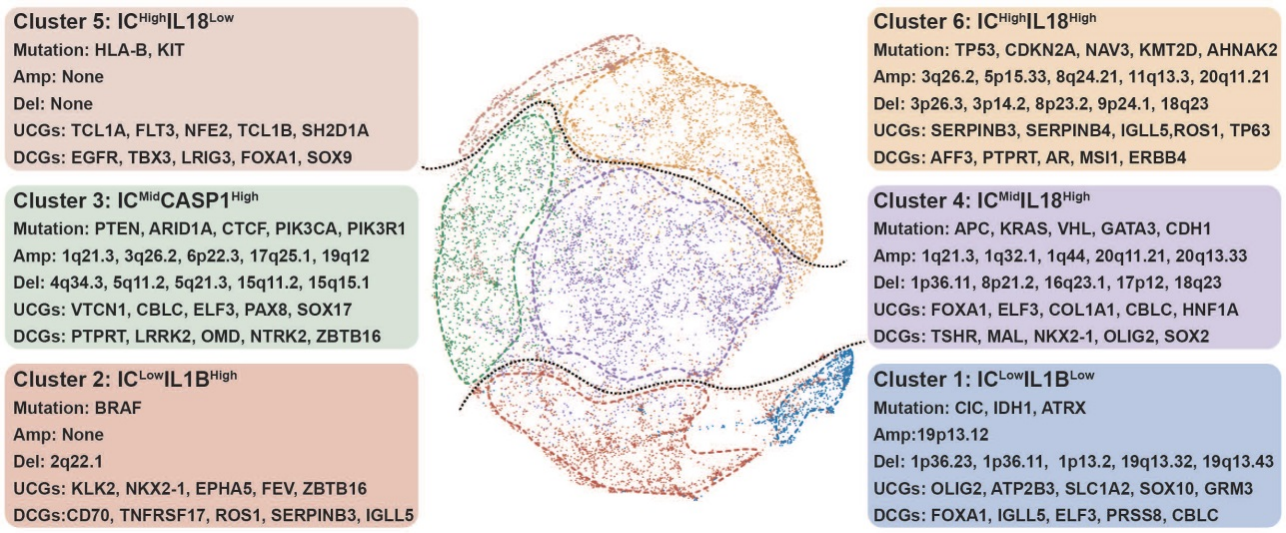
** Genomic determinants of inflammasome clusters.** The layout of TumorMap was conducted from sample Euclidean similarity based on the five inflammasome-signaling-related scores, and similar samples were grouped. Distinct genomic features (Mutation, SCNA, and dysregulated coding genes) are also depicted. Differential enrichment or deletion of SCNA drivers, mutation drivers, and coding mRNAs in each cluster compared to all other clusters is shown. IC, inflammasome complex; SCNA, somatic copy-number alteration; UCG, upregulated coding gene; DCG, downregulated coding gene.

*IC^Low^IL1B^Low^* tumors had high mutation frequencies of *CIC, IDH,* and *ATRX*, along with high frequencies of 1p/19q codeletion, consistent with the cluster 1-enriched LGG (Figure 3, Figure S6, Figure S7A and D) 33. A recent study suggested that IDH mutation was associated with reduced inflammatory response 34. On the other hand, as one of the top upregulated cancer genes in cluster 1 (Figure 3, Figure S8), ATP2B3 was reported to maintain the cellular homeostasis of Ca (2^+^), thus suppressing the activation of NLRP3 and NLRC5 inflammasomes 35, 36. Consistently, tumors with high expression of ATP2B3 were found to be with attenuated expression of NLRP3 and NLRC5 (Figure S9). Therefore, ATP2B3 might serve as a major contributor to the inflammasome suppression in cluster 1, which should be experimentally investigated. In comparison with other clusters, cluster 1 was found to have typical downregulation of miRNAs (Figure S8). MiR-200 family was identified as the top down-regulated miRNAs in cluster 1 (Table S8), which had been reported to facilitate tumor inflammation.

#### Cluster 2

*IC^Low^IL1B^High^* tumors displayed high-frequency BRAF mutation (Figure 3). Drug sensitivity analysis showed that cluster 2 tumors were resistant to BRAF-targeted drugs (Figure S10, Table S8), consistent with the characteristic of tumors resistant to BRAF inhibitors had low enrichment of proinflammatory genes 37. Among the top enriched cancer genes in cluster 2 (Figure 3), *ZBTB16* can inhibit inflammatory response 38, resulting in a low inflammasome score in this cluster. Interestingly, the top three down-regulated miRNAs in this cluster all participated in regulating inflammatory response (Table S8) 39. Furthermore, tumors in clusters 1 and 2 had a low degree of genomic instability (Figure S7B-C), because of which these two clusters had suppressed IC scores.

#### Cluster 3

*IC^Mid^CASP1^High^* tumors were enriched with high mutation frequencies in *PTEN, ARID1A, CTCF, PIK3CA,* and* PIK3R1* (Figure 3, Figure S6). PTEN deficiency can trigger inflammatory response 40. Moreover, *ARID1A* and *PIK3CA* mutations usually coexist to enhance a pro-tumorigenic inflammatory response 41, 42.

#### Cluster 4

*IC^Mid^IL18^High^* tumors were featured by high mutation of *APC, KRAS, VHL, GATA3,* and *CDH1* (Figure 3, Figure S6). *APC* and *CDH1* can regulate inflammatory response by destabilizing PECAM-1 43. Interestingly, clusters 3 and 4 tumors share some features, including an increased number of segments, a high aneuploidy score, higher frequency of 1q21.3 deletion, and increased transcription of *CBLC* and *ELF3* (Figure 3, Figure S7B-D, Figure S8). A previous report showed that ELF3 could activate NLRP3 inflammasome by suppressing MARK4 promoter activity 44, indicating that similar mechanisms to enhance inflammasome signaling are at work in these two clusters.

#### Cluster 5

*IC^High^IL18^Low^* tumors showed increased mutation frequencies of HLA-B and KIT with no significant alteration in the focal region, along with decreased number of segments (Figure 3, Figure S6, Figure S7B and D). Based on previous studies, the main genomic and epigenetic features enriched in this cluster are correlated with hematological malignancies (Figure 2C) 45-48.

#### Cluster 6

*IC^High^IL18^High^* tumors contained high mutation frequencies of *TP53, CDKN2A, NAV3, KMT2D,* and *AHNAK2* (Figure 3, Figure S6). The mutation of *TP53* can promote aberrant inflammation in glioblastoma 49. Moreover, this cluster tumor is associated with amplified *TERC, TERT, MYC, CCND1,* and *BCL2L1* (Figure 3, Figure S7D). The balance between the levels of c-Myc and TP53 plays a crucial role in regulating inflammatory response 50, 51. Meanwhile, TERC as an RNA component of telomerase can promote an inflammatory response in a telomerase-independent manner 52. SERPINB3/4 and TP63, which are among the top upregulated, can activate inflammation (Figure 3, Figure S8) 53, 54. Therefore, we hypothesize that TP53 mutation, along with the amplification of MYC and telomerase-related genes, closely correlates with elevated inflammasome signaling in cluster 6 tumors by up-regulating SERPINB3/4 and TP63.

### IC^Mid^ and IC^High^ clusters exhibited immunosuppressive phenotypes

**Figure 4:**
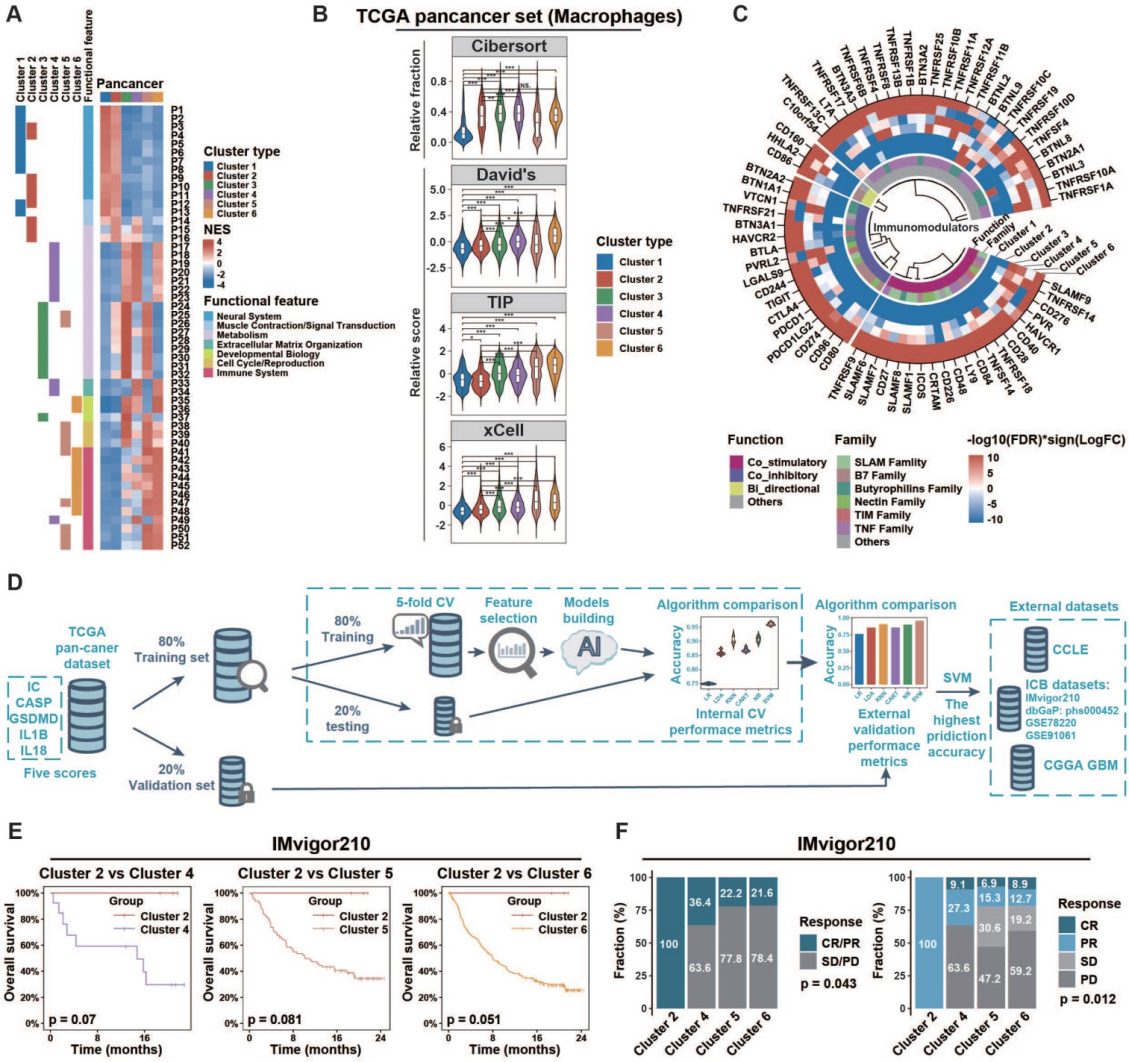
** Tumors of different inflammasome clusters have a distinct tumor immune microenvironment and response to checkpoint blockade. (A)** Functional enrichment analyses of each cluster compared to all other clusters in pan-cancer were conducted using the GSEA function in clusterProfiler R package. The pathways with adjusted p < 0.01 and top ten enrichment in each cluster are shown in the heatmap. The NES value is represented by the color intensities. Row annotation on the left indicates functional features of the related pathways (described in Table S9) and enrichments in related clusters. **(B)** The distribution of macrophage score calculated from different gene sets across clusters. **(C)** Profile difference of immunomodulators between one cluster and all other clusters was assessed by Wilcoxon rank-sum test. Color intensities represent negative log10(FDR) multiplied by the sign of the logFC. **(D)** Workflow of prediction model construction. Briefly, TCGA samples were split randomly into training (80%) and validation (20%) sets. The training set was then used to compare the prediction accuracies of six algorithms by five-fold cross-validation process to overcome overfitting. The accuracies of six algorithms were further assessed using the validation set as an external-layer evaluation. Then, SVM with the highest prediction accuracy (96%; Table S3) was applied to external datasets (CCLE, CGGA GBM, IMvigor210, dbGaP: phs000452, GSE78220, and GSE91061). **(E)** Overall survival of cases with metastatic urothelial cancer from IMvigor210CoreBiologies clinical trial was compared among clusters 2/4/5/6. The log-rank test was used to calculate the p-value. Clusters 1/3 were not identified by the SVM algorithm in this clinical trial cohort. **(F)** The difference of clinical response to immunotherapy among clusters was assessed using the chi-square test. NES, normalized enrichment score; CV, cross-validation; SVM, support vector machine; Cibersort, macrophage related gene set from Cibersort website (https://cibersort.stanford.edu/); David's, macrophage related gene set from David's study; TIP, macrophage related gene set from Tumor Immunophenotype (http://biocc.hrbmu.edu.cn/TIP/); xCell, macrophage related gene set from xCell website (https://xcell.ucsf.edu/); NS, not significant; *p < 0.05, **p < 0.01, ***p < 0.001.

To explore the biological phenotype among inflammasome clusters, we first conducted GSEA analysis to identify cluster-related gene sets and pathways. At the pan-cancer level (Figure 4A), clusters 1 and 2 were mainly associated with increased expression of neural system processes; 3 and 4 with metabolism and extracellular matrix organization; and 5 and 6 with immune system process. Similar patterns were also observed at the cancer-specific level (Figure S11, Table S9). Specifically, based on the distribution of scores calculated from nonredundant gene programs, pathway signatures for drug targets, and canonical pathways across various clusters, we found that immune related gene sets such as GP2_Inmmune-T cell/B cell, GP11_Immune-IFN, PD1_signaling, and CTLA4_pathway (Figure S12A-B, Table S6) exhibited consistently enhanced enrichment in IC^Mid^ and IC^High^ clusters rather than IC^Low^ ones. Then, we profiled the anti-tumor immunity cycle based on the Tumor Immunophenotype (TIP) algorithm 25. The initial (steps 1 and 2) and activation (steps 3, 4, and 5) phases of the anti-tumor immunity cycle were significantly enhanced in clusters with inflammasome signaling activation (clusters 3/4/5/6); whereas the effective anti-tumor immunity dampened at steps 6 (tumor cell recognition by T cell) and 7 (killing of cancer cell) (Figure S12C, Table S6). Moreover, according to the Tumor Immune Dysfunction and Exclusion (TIDE) algorithm 55, T cells in IC^Mid^ and IC^High^ clusters tended to be dysfunctional (Figure S13). Consistently, IC^Mid^ and IC^High^ clusters showed increased immunosuppressive scores calculated by the method described previously (Figure S14A lower panel, Table S6) 56. Together, these findings suggest that clusters with inflammasome signaling activation exhibited an immunosuppressive phenotype.

### Extrinsic and intrinsic mechanisms of the immunosuppressive phenotype in IC^Mid^ and IC^High^ clusters

Based on the immunoediting theory 57, both extrinsic and intrinsic mechanisms may contribute to the distinct immune status of tumors. Here, we first evaluated the microenvironmental composition to identify the potential extrinsic mechanisms. We found that IC^Mid^ and IC^High^ clusters had higher immune score but lower tumor purity than IC^Low^ clusters (Figure S14A, Table S6). Moreover, immune effector cells, such as cytotoxic cells and CD8^+^ T cells, along with immunosuppressive cells such as macrophages and Tregs, were more enriched in IC^Mid^ and IC^High^ clusters. CIBERSORT algorithm was used to evaluate the fractions of immune cells. Macrophages were the major immunosuppressive cellular component in IC^Mid^ and IC^High^ clusters (Figure S14B), which could be validated by three well-established gene sets 23, 25, 26 and IHC score from GSE39582 (Figure 4B, Figure S14C). Furthermore, higher expression of M2-polarization markers [CD200R1, CD163, CD206 (also named MRC1), TLR1, and TLR8] 58, 59 coupled with lower expression of M1-polarization markers [NOS2 (also named iNOS) and TLR4] 60, 61 were observed in IC^Mid^ and IC^High^ clusters, suggesting that these macrophages mainly played an immunosuppressive role (Figure S14D). Overall, macrophages enrichment might be an extrinsic factor in maintaining the immunosuppression in IC^Mid^ and IC^High^ clusters.

Subsequently, we focused on the expression of the immunomodulator including costimulatory and coinhibitory molecules, which was another main driving force in regulating the intrinsic immune escape 57. Both costimulatory and coinhibitory molecules were relatively upregulated in clusters 3/4/5/6 (Figure 4C, Table S10), which indicates T-cell dysfunction according to the tidal model theory 62. These results suggest that aberrant expression patterns of immunomodulators might be an intrinsic contributor to the immunosuppression in IC^Mid^ and IC^High^ clusters.

### IC^Mid^ and IC^High^ tumors show therapeutic resistance to ICB regimens

Mounting evidence suggest that tumor-associated macrophage (TAM) infiltration and overexpression of immune checkpoints dampened the therapeutic response to several types of immunotherapies. These encouraged us to explore whether inflammasome clusters correlate with the clinical outcome of ICB therapy. Firstly, we sought to develop effective approach to group external tumor samples based on our inflammasome clustering system (Figure 4D, Methods). Six machine-learning algorithms were developed and Support Vector Machine (SVM) algorithm with the highest validity was selected for further analysis (Figure 4D, Table S3). By SVM classification, patients who received anti-PD-L1 treatment from the IMvigor210 immunotherapy cohort were grouped into clusters 2/4/5/6 but not clusters 1/3 (Table S5C). The pattern of inflammasome signaling steps and macrophage enrichment across inflammasome clusters in this clinical cohort was similar to that of the TCGA cohort, verifying the validity of the SVM method (Figure S15A‒B). Although the log-rank test results failed to reach statistical significance, IC^Mid^ and IC^High^ clusters showed reduced survival time compared to IC^Low^ counterparts (Figure 4E). Furthermore, IC^Mid^ and IC^High^ tumors achieved less clinical benefit from anti-PD-L1 treatment than IC^Low^ ones (Figure 4F). Additionally, cutaneous melanoma patients who received anti-PD1 therapy from three cohorts (dbGaP: phs001036, GSE91061, and GSE78220) were used to further explore the ICB therapy response across inflammasome clusters. After SVM based inflammasome classification, we found that IC^Mid^ and IC^High^ tumors tended to suffer resistance to anti-PD1 therapy (Figure S15C, Table S5D). Together, these findings suggest that clusters with inflammasome signaling activation were resistant to ICB regimens.

### Inflammasome inhibition suppresses TAM infiltration and PD-L1 expression in tumors with high inflammasome signaling activity

Subsequently, we sought to validate the relationship between inflammasome signaling and tumor immunosuppression. TCGA-GBM and TCGA-BRCA were selected as representative tumor types for this purpose. Parallel analyses were also conducted in our in-house CGGA-GBM RNAseq cohort for external validation. The distribution of five inflammasome-signaling-related scores in TCGA-GBM, TCGA-BRCA, and CGGA-GBM was consistent with scores of pan-cancer analyses (Figure S16, Table S6). Patients in IC^Mid^ and IC^High^ clusters had a reduced survival time compared with those in the IC^Low^ cluster (Figure S17). Furthermore, we found that immunosuppressive biological programs were significantly enhanced in clusters with inflammasome activation (Figure S16, Figure S18A, Table S6). Specifically, both extrinsic macrophage infiltration and intrinsic immunomodulator expression were increased in IC^Mid^ and IC^High^ clusters (Figure S16B-D, Figure S18B-C, Table S6, Table S11). Together, GBM and BRCA showed distinct inflammasome phenotype similar with that identified from pan-cancer analysis, suitable for further analysis as representative tumor types.

**Figure 5:**
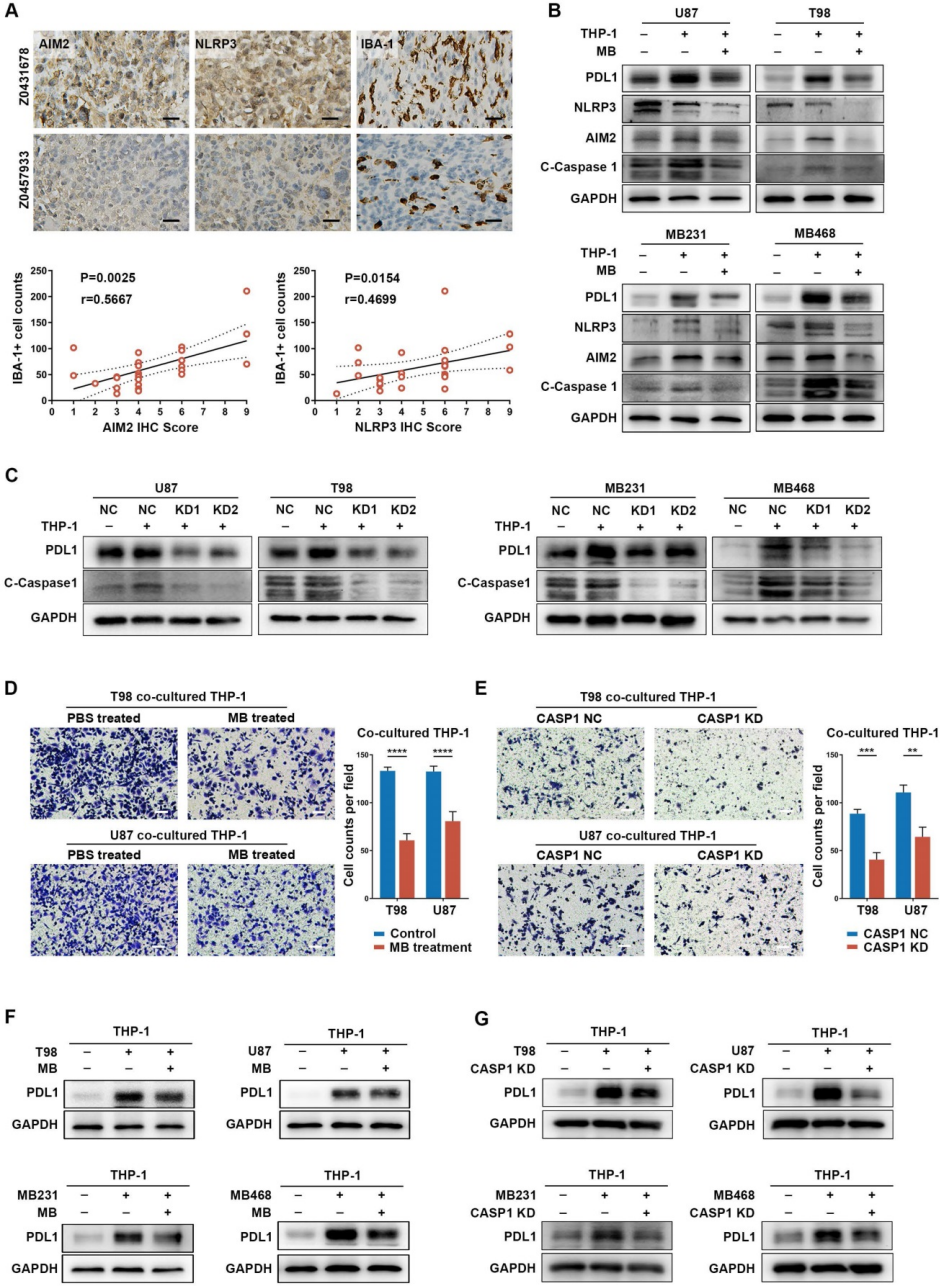
** Inflammasome signaling plays important roles in cancer cells and TAM interaction. (A)** Immunohistochemistry and quantitative correlation analyses of AIM2 and NLRP3 expression and IBA-1^+^ TAM infiltration in GBM patients. Scale bar: 25 μm. **(B and C)** Western blotting analysis of the inflammasome (NLRP3 and AIM2), C-Caspase 1 (cleaved caspase 1), and PD-L1 expression in THP-1 co-cultured GBM and BRCA cells, pre-treated with MB (B) or CASP1 targeted siRNA (C). **(D and E)** Migration analysis of THP-1, co-cultured with cancer cells that were pre-treated with MB (D) or CASP1 targeted siRNA (E). Scale bar: 25 μm. **(F and G)** Western blotting analysis of PD-L1 expression in THP-1 which was co-cultured with cancer cells pre-treated with MB (F) or CASP1 targeted siRNAs (G). **p < 0.01, ***p < 0.001, ****p < 0.0001.

Then, we sought to verify the possible role of inflammasome signaling in cancer cells and TAM interaction. It is widely accepted that the expression level of NLRP3 and AIM2 can predict the inflammasome signaling activity in cancers 63. Therefore, NLRP3 and AIM2 were chose to be detected in in-house GBM samples and publicly available IHC data of colon cancer patients 64. We found that patients with higher AIM2 and NLRP3 expression tended to have higher TAM (IBA-1^+^) infiltration (Figure 5A, Figure S19A), which indicates positive relation between inflammasome signaling and TAM infiltration. Next, GBM cell lines [U87M (cluster 4) and T98 (cluster 4)] and BRCA cell lines [MB231 (cluster 4) and MB468 (cluster 3)] were used for cellular experiments (Table S5B). When cancer cells were co-cultured with TAM (Figure S19B), we found that the expression of NLRP3, AIM2, cleaved-caspase 1, and IL-1β was significantly upregulated (Figure 5B-C, Figure S19C-D). Moreover, expression of the most widely studied immune checkpoint, PD-L1, was increased in TAM co-cultured cancer cells (Figure 5B-C). These results suggest a mechanism of TAM-mediated inflammasome signaling activation and immune invasion ability upregulation in cancer cells.

Methylene Blue, a broad-spectrum inflammasome inhibitor 65, and CASP1 siRNAs were used to suppress inflammasome signaling activation, reflected by decreased IL-1β expression, in cancer cells (Figure S20A-C, Figure S21A-B). Interestingly, inflammasome inhibition had a remarkable negative effect on PD-L1 expression in cancer cells (Figure S20A and C). Moreover, when TAM-mediated inflammasome signaling enhancement in cancer cells was abrogated by inflammasome inhibition, TAM-promoted PD-L1 expression in cancer cells was also decreased (Figure 5B-C, Figure S19C-D). These results suggest an important role of inflammasome signaling in regulating immune invasion ability of cancer cells even under a cancer cell-TAM interaction context.

Subsequently, to clarify the role of inflammasome signaling in cancer cell-mediated TAM's biological phenotype, we co-cultured inflammasome signaling suppressed cancer cells with TAM. Results showed that inflammasome suppression in cancer cells significantly decreased TAM infiltration, accompanied by an attenuated tendency to immunosuppressive M2 polarization (Figure 5D-E, Figure S22A-C). Moreover, inflammasome inhibition also repressed the effect of cancer cell-induced PD-L1 overexpression in TAM (Figure 5F-G), suggesting a decreased cancer cell-promoted immunosuppressive ability of TAM. Together, these results indicate a critical role of inflammasome signaling in regulating the cancer cells and TAM interaction.

### Inflammasome inhibition amplifies the curative effect of PD-L1 blockade in inflammasome highly activated tumors

**Figure 6:**
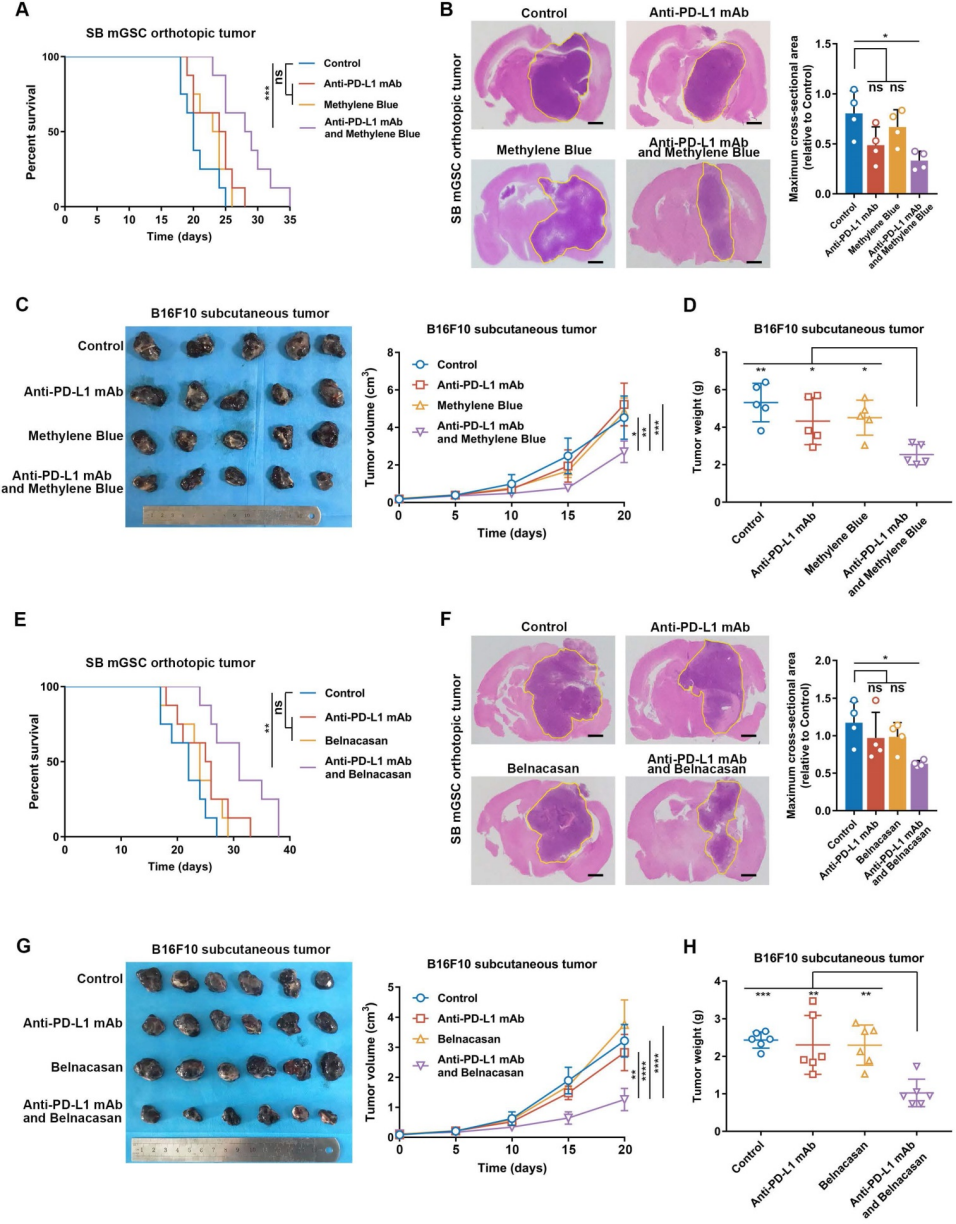
** Inflammasome inhibition increases the efficacy of PD-L1 blockade in GBM and melanoma models. (A and E)** Survival analysis of GBM-bearing mice. **(B and F)** Hematoxylin and eosin staining and the quantification of the relative maximum cross-sectional area of orthotopic GBM tumors. Scale bar: 500 μm. **(C and G)** Images and the growth rate of B16F10 melanoma tumor bulks. **(D and H)** The weight of the B16F10 melanoma tumor bulks immediately after the tumor enucleation. *p < 0.05, **p < 0.01, ***p < 0.001, ****p < 0.0001.

To explore the relation between inflammasome activity and ICB therapeutic efficacy, we performed PD-L1 blockade therapy in SB mGSC and GL261 cell line orthotopic GBM models and B16F10 cell line subcutaneous melanoma model. SB mGSC and B16F10 were classified as cluster 5*,* but GL261 was classified as cluster 2 (Table S5E). Consistent with previous findings 66, the GL261 GBM model showed a better response to PD-L1 blockade, reflected in significantly prolonged survival time, decreased tumor volume, and increased CD4^+^ and CD8^+^ T cells infiltration (Figure S23A-C). In contrast, PD-L1 blockade elicited negligible curative effect in SB or B16F10 tumor models (Figure 6). However, when we combined MB treatment with PD-L1 blockade (Figure S24A, Figure S25A), the efficacy of PD-L1 blockade was significantly improved. Results showed that combined treatment prolonged survival time and suppressed tumor growth in both SB and B16F10 tumors (Figure 6A-D). Moreover, combined treatment significantly inhibited cell proliferation but increased cell apoptosis (Figure S24B, Figure S25B). We also combined Belnacasan, a CASP1 specific inhibitor that effectively reduced the cleaved-caspase1 and IL-1β expression in tumors (Figure S26A-B), with PD-L1 blockade. Similar results were achieved that combined treatment prolonged survival time and inhibited tumor growth of both tumor models (Figure 6E-H).

By analyzing immune cell constituents, we found PD-L1 blockade alone could not effectively increase the infiltration of adaptive immune cells (CD8^+^ and CD4^+^ T cells) or their anti-tumor functions comparing with those of control ones (Figure S24C-E, Figure S25C-E). However, when inflammasome inhibition was combined to PD-L1 blockade, the infiltration of CD4^+^ T cell and CD8^+^ T cell was significantly increased, accompanied by an increased proportion of Th1 and functional CD8^+^ T cells (Figure S24C-E, Figure S25C-E). Moreover, the combined treatment effectively suppressed TAM infiltration (Figure S24C, Figure S25C) and its M2 subtype polarization (Figure S24F, Figure S25F) compared with the control group. Together, these results suggest that inflammasome inhibition increases the therapeutic efficacy of PD-L1 blockade by improving the anti-tumor immune environment in inflammasome highly activated tumors.

### The Cancer Analysis of Inflammasome Balance (CAIB) website

Thus, we established a CAIB website (http://l.neuroscience.org.cn/) to classify the newly uploaded transcriptomic profiles of patients into inflammasome clusters. In-house glioma dataset from the CGGA GBM cohort and the clusters predicted by SVM algorithm based on these five inflammasome-signaling-related scores showed similar biological features compared with that from the TCGA GBM cohort (Figure S16A and D, Figure S17, Figure S18), thereby confirming the accuracy and applicability of our proposed classification system.

## Discussion

**Figure 7:**
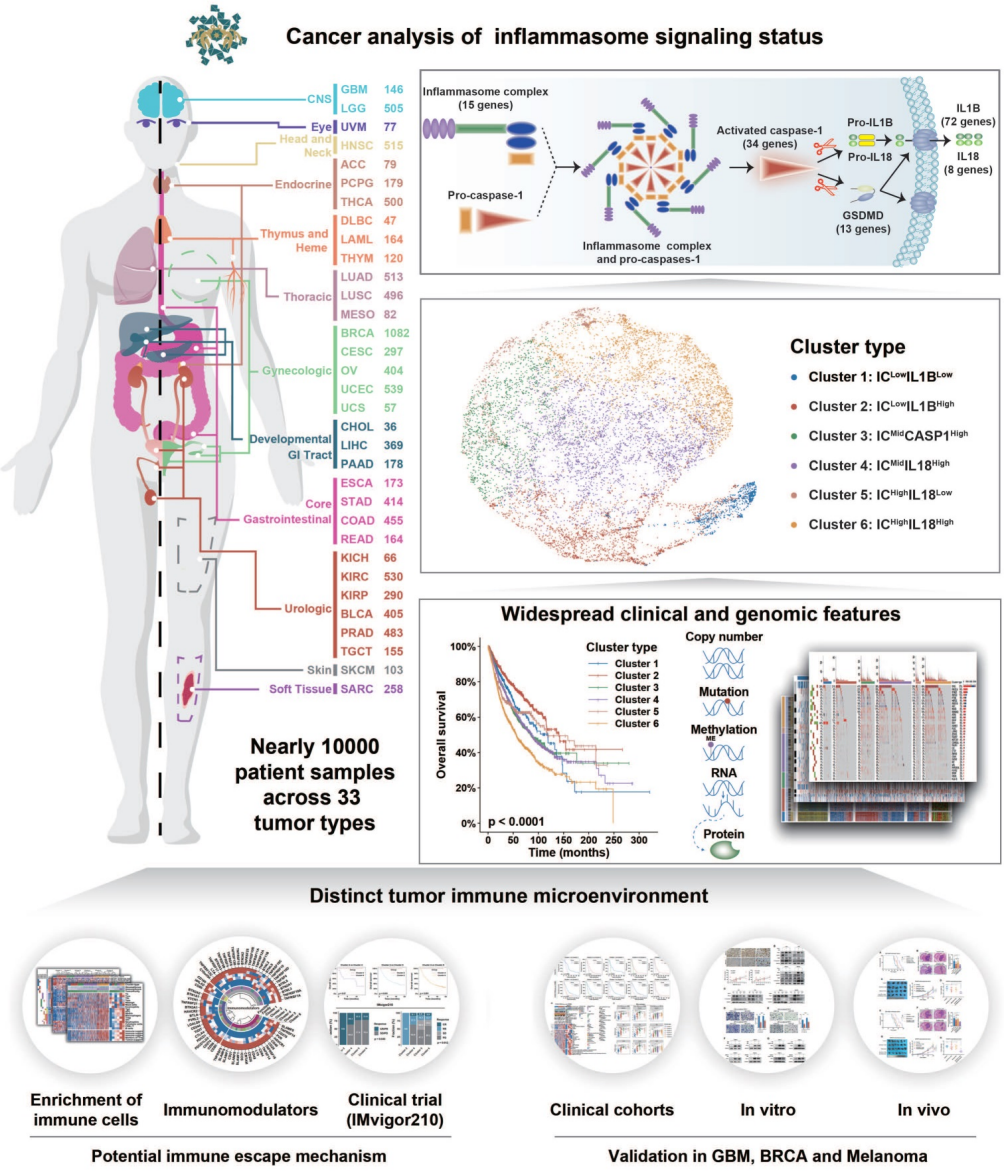
** Schema of multi-omic analysis of the inflammasome signaling status across 33 tumor types.** Nearly 10000 patient samples across 33 tumor types were collected from TCGA to investigate inflammasome signaling status based on five gene sets representing the sensing, activation, and termination steps of the inflammasome signaling. Six inflammasome clusters were robustly established with distinct molecular, biological, clinical, and therapeutic features, which was validated in external cohorts and *in vitro* and vivo experiments.

Inflammasome signaling plays an important role in tumorigenesis and tumor immunosuppression 67. Therefore, inflammasome signaling needs to be clarified to establish an accurate classification system to guide individualized therapeutic strategies. To the best of our knowledge, this is the first systemic analysis on inflammasome heterogeneity based on a large patient cohort. Our study revealed the comprehensive molecular features and biological functions of inflammasome signaling across 9881 samples of 33 tumor types (Figure 7). Overall, our study resulted in three key findings: (1) establishment of inflammasome classification system with distinct molecular, biological, and clinical features; (2) identification of the positive relationship between inflammasome signaling and macrophages infiltration and M2 polarization; and (3) improved therapeutic efficacy of ICB regimen combined with inflammasome suppression in tumors with activated inflammasome signaling.

Tumor classification is important to understand tumors and improve the outcome of anti-tumor therapy. Recent studies have shown that the activation of inflammasome signaling correlated with clinical outcome and mediated resistance to various cancer regimens 68, 69. Our study is the first to propose a rational stratification of tumor patients based on five inflammasome-signaling-related scores representing different stages of inflammasome signaling. Different inflammasome clusters exhibited distinct molecular, biological, and clinical features, which may be a benefit for conducting personalized therapeutic interventions in each cluster. Firstly, miR-200 family was reported to enhance sensitivity to chemotherapy and radiotherapy 70. We found that *Cluster 1: IC^Low^IL1B^Low^* tumors were characterized by the low expression of miR-200 family. Therefore, over-expressing the miR-200 family may improve clinical outcomes in cluster 1. Secondly, BRAF mutation enriched in *Cluster 2: IC^Low^IL1B^High^* tumors were identified to be resistant to BRAF-targeted drugs, indicating that patients in this cluster will receive less benefits from BRAF inhibitors. Finally, *Cluster 6: IC^High^IL18^High^* tumors with the worst survival prognosis were characterized with a higher amplified frequency of MYC than other clusters, suggesting that therapies targeting MYC may extend the survival time of patients in this cluster. Together, our tumor classification method based on inflammasome signaling can define tumor subtypes regardless of tumor lineage-specific markers or patterns, thus allowing an evaluation of personalized targeting therapy in patients with all types of tumors.

Rather than working alone, cancer cell relies on the tumor microenvironment (TME) to facilitate the protection from host immunosurveillance 71. The non-tumor cells, including neutrophils, MDSCs, and macrophages play a crucial role in immunosuppressive TME formation. A previous study showed that tumor inflammasomes play a key role in tumor suppression by recruiting neutrophils 72. However, most studies support a pro-tumor role for neutrophils. Furthermore, inflammasomes also could enhance the enrichment of MDSCs and TAMs in TME 18. In this study, we observed similar patterns that cluster with inflammasome signaling activation were identified with enhanced enrichment of neutrophils, MDSCs, and macrophages (Figure S12C, Figure S14A). Among these immune cells, we found that macrophages were the major immune cell population enriched in IC^Mid^ and IC^High^ clusters. Additionally, there was a positive loop between the cancer cells and TAM in activating inflammasome signaling. Inflammasome inhibition impaired TAM recruitment and suppressed its M2 polarization, implying the potential of inflammasome inhibition in TAM modulating therapy. However, further explanation is still needed to clarify how the inflammasome signaling regulates TAM infiltration and polarization.

Although ICB therapy is widely used in various malignant diseases, only a few patients get clinical benefits. Until now, several studies have indicated a connection between ICB responders and the expression of inflammasome signatures 73. However, whether inflammasomes activation is beneficial to anti-PD-1 therapy. In this study, we found that patients of IC^Mid^ and IC^High^ have the worst response to ICB treatment, caused by the immunosuppressive microenvironment and immune checkpoint dysregulation. Inflammasome inhibition in IC^Mid^ and IC^High^ models would significantly amplify the efficacy of PD-L1 blockade. Moreover, to widen the application of our findings, a tumor classification website, CAIB, was established so that other researchers can employ our inflammasome classification method. Therefore, our study provides a practical approach for inflammasome-based tumor classification and treatment guidance.

## Conclusion

In summary, we identified inflammasome classification system with important biological and clinical implications, which would be helpful for personalized therapeutic strategies. The main advantage of our research is derived from the large sample-sized tumor cohort, a multidimensional profiling of inflammasome signaling, and the combination of bioinformatic and experimental methods. However, further investigation of the molecular mechanisms underlying inflammasome signaling in modulating immunosuppression is required. Overall, our study showed that inflammasome signaling status across tumor types may help develop effective individual therapeutic interventions.

## Supplementary Material

Supplementary figures.

Supplementary table 1: Inflammasome-signaling-related genes.

Supplementary table 2: Sample numbers from different datasets.

Supplementary table 3: Classification prediction accuracy of six machine learning algorithms by python language.

Supplementary table 4: Enrichment or deletion of inflammasome-signaling-related gene sets and differential expression of 141 inflammasome-signaling-related genes.

Supplementary table 5: Identification of inflammasome clusters.

Supplementary table 6: Distribution of each score across inflammasome clusters.

Supplementary table 7: Enrichment of tumor type in inflammasome clusters (related to figure 2C).

Supplementary table 8: Genomic features differentially altered in each inflammasome cluster compared to all other clusters.

Supplementary table 9: GSEA results (NES value) of each cluster at pan-cancer or cancer-sepecific level (related to figure 4A and figure S11).

Supplementary table 10: Differential expression analysis of immunomodulators between one cluster and all other clusters (related to figure 4C).

Supplementary table 11: Differential expression analysis of immunomodulators across clusters in GBM and BRCA.

Supplementary table 12: Target gene symbols and corresponding sequences of primers.

## Abbreviations

ACC: Adrenocortical carcinoma
BH: Benjamini and Hochberg
BLCA: Bladder Urothelial Carcinoma
BRCA: Breast invasive carcinoma
CAIB: the cancer analysis of inflammasome balance
CART: Classification and Regression Trees
CASP1: caspase-1
CCLE: the Cancer Cell Line Encyclopedia
CESC: Cervical squamous cell carcinoma and endocervical adenocarcinoma
CGGA: the Chinese Glioma Genome Atlas
CHOL: Cholangiocarcinoma
COAD: Colon adenocarcinoma
DEGs: differential expression genes
DLBC: Lymphoid Neoplasm Diffuse Large B-cell Lymphoma
ESCA: Esophageal carcinoma
ESTIMATE: Estimation of STromal and Immune cells in MAlignant Tumor tissues using Expression data
FDR: false discovery rate
GBM: Glioblastoma multiforme
GDSC: Genomics of Drug Sensitivity in Cancer
GSDMD: Gasdermin D
HNSC: Head and Neck squamous cell carcinoma
HR: hazard ratios
IC: inflammasome complex
ICB: immune checkpoint blockade
IHC: immunohistochemical
KEGG: Kyoto Encyclopedia of Genes and Genomes
KICH: Kidney Chromophobe
KIRC: Kidney renal clear cell carcinoma
KIRP: Kidney renal papillary cell carcinoma
KM: Kaplan-Meier
KNN: KNeighbors Classifier
LAML: Acute Myeloid Leukemia
LDA: Linear Discriminant Analysis
LGG: Brain Lower Grade Glioma
LIHC: Liver hepatocellular carcinoma
lncRNA: long non-coding RNA
logFC: log2(fold change)
LR: Logistic Regression
LUAD: Lung adenocarcinoma
LUSC: Lung squamous cell carcinoma
MAD: maximum absolute deviation
MB: methylene blue
MESO: Mesothelioma
mGSC: mouse glioma sphere cell
NB: Gaussian NB
NES: Normalized Enrichment Score
OV: Ovarian serous cystadenocarcinoma
PAAD: Pancreatic adenocarcinoma
PAMPs or DAMPs: pathogen- or danger-associated molecular patterns
PBMC: peripheral blood mononuclear cell
PCPG: Pheochromocytoma and Paraganglioma
PRAD: Prostate adenocarcinoma
READ: Rectum adenocarcinoma
RPPA: Reverse Phase Protein Array
SARC: Sarcoma
SB: sleeping beauty
SCNA: somatic copy-number alteration
SKCM: Skin Cutaneous Melanoma
ssGSEA: single sample gene set enrichment analysis
STAD: Stomach adenocarcinoma
SVM: support vector machine
TAM: tumor-associated macrophage
TCGA: The Cancer Genome Atlas
TGCT: Testicular Germ Cell Tumors
THCA: Thyroid carcinoma
THYM: Thymoma
TIDE: Tumor Immune Dysfunction and Exclusion
TIP: Tumor Immunophenotype
TME: tumor microenvironment
TMTI: tumor microenvironment immune type
Tregs: regulatory T cells
TSS: transcription start site
UCEC: Uterine Corpus Endometrial Carcinoma
UCS: Uterine Carcinosarcoma
UCSC Xena: University of California, Santa Cruz Xena
UVM: Uveal Melanoma
