# Supplementary material for: Establishment of tumor inflammasome clusters with distinct immunogenomic landscape aids immunotherapy
Source: Theranostics. 2021 Oct 17;11(20):9884–903. doi: 10.7150/thno.63202 (PMC8581407; doi:10.7150/thno.63202)
Supplement: Supplementary file 1 — Supplementary figures. [file thnov11p9884s1.pdf]

Supplementary figures

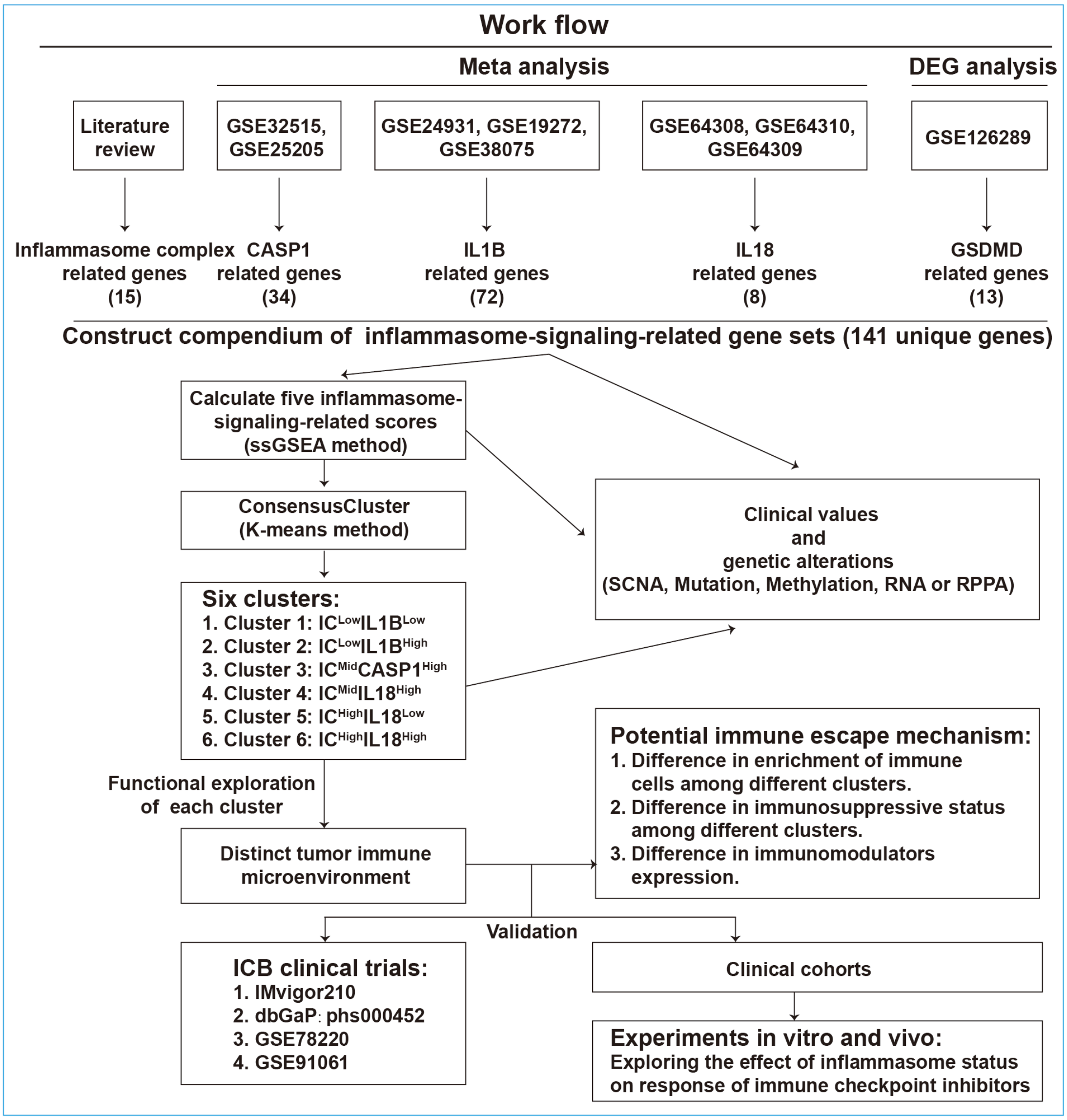

Figure S1. Workflow of our study.

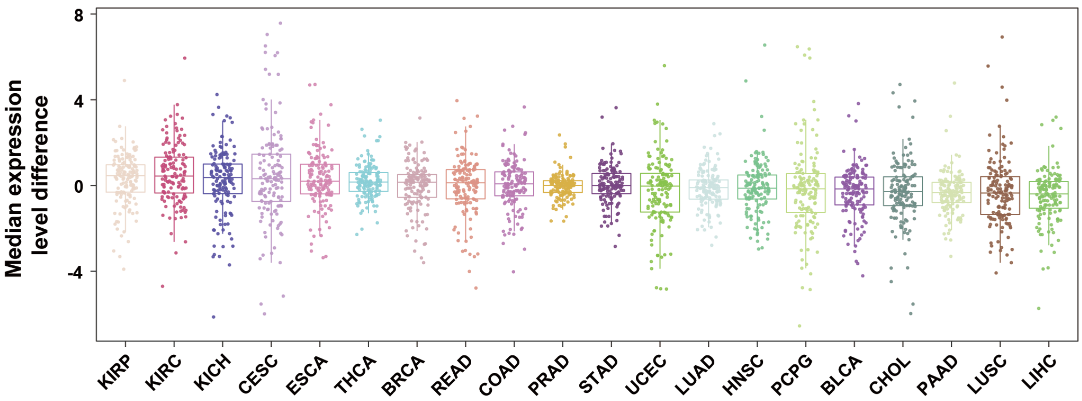

Figure S2. Median expression level difference of 141 inflammasome-signaling-related genes between tumor and non-tumor tissue across 20 tumor types.

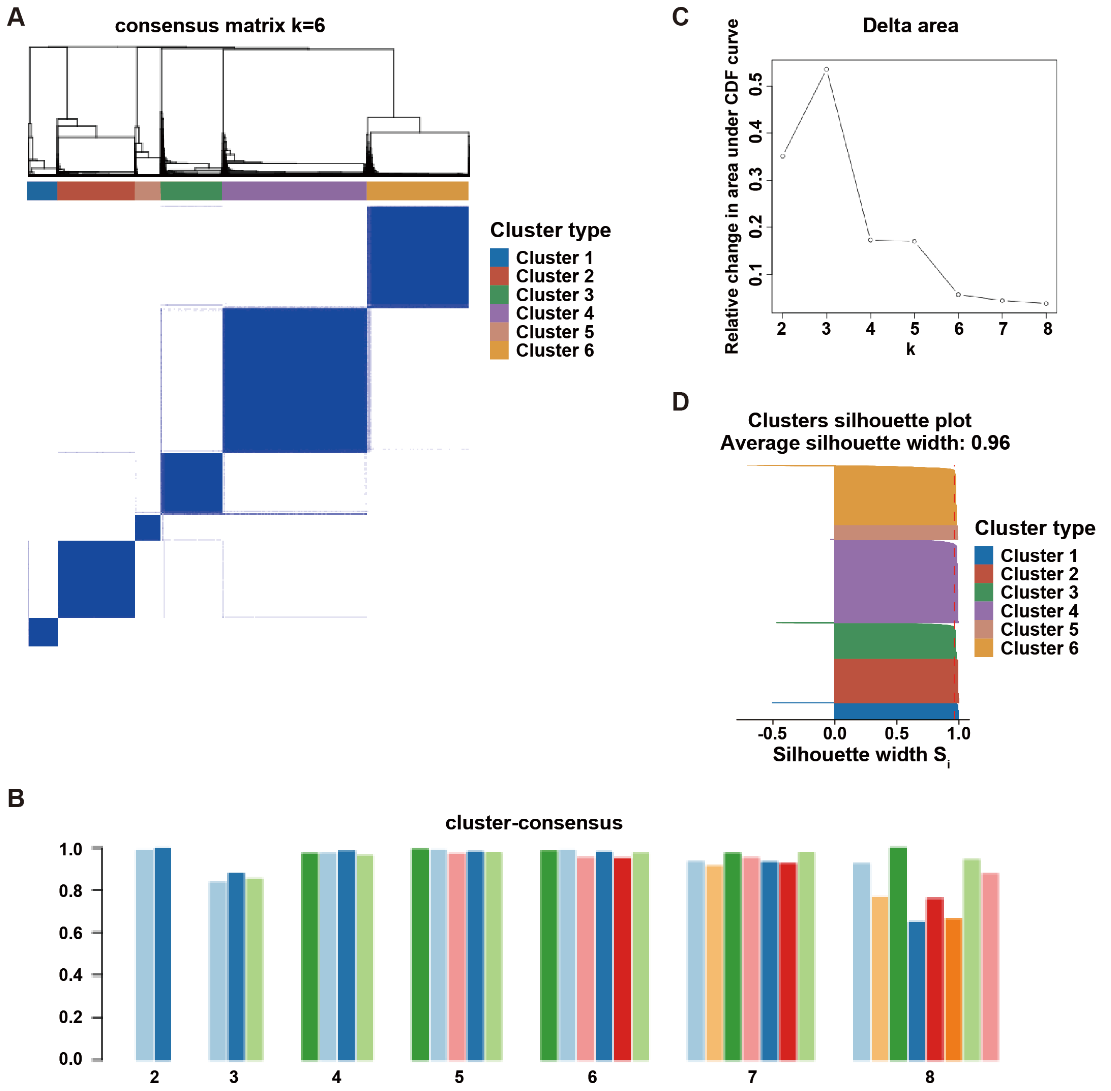

Figure S3. Identification of inflammasome clusters. (A) Consensus clustering matrix of TCGA pan samples for k = 6. (B) Consensus clustering stability is depicted in Cluster-Consensus plot. High values indicate a cluster with high stability and low values indicate a cluster with low stability. (C) Delta area plot shows the relative change in area under the Consensus Cumulative Distribution Function (CDF) curve comparing k and k-1, which assisted in determining the optimal clustering numbers. (D) Silhouette analysis of inflammasome-signaling-related clustering.

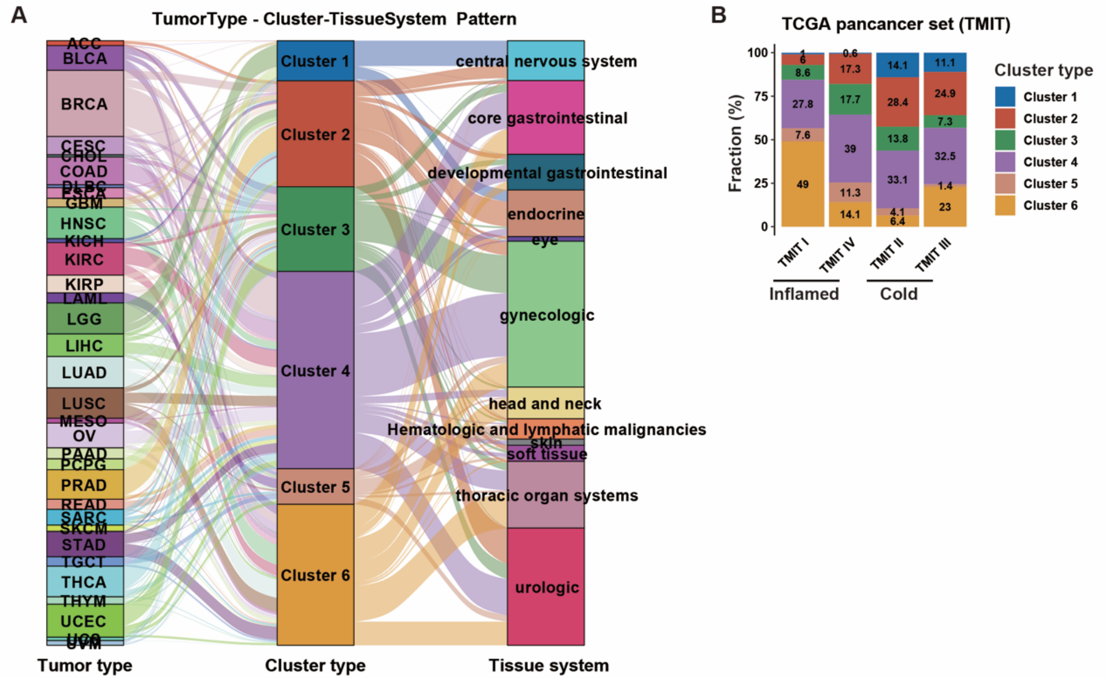

Figure S4. Distribution of inflammasome clusters. (A) Correlation of TCGA tumor types, inflammasome clusters, and tissue systems. The Sankey diagram shows the tumor-type composition of each cluster. The types of tissue system are shown on the right. (B) The distribution of inflammasome clusters across immunogenic/inflamed (TMIT I and IV) and cold tumors (TMIT II and III). TMIT, tumor microenvironment-immune type.

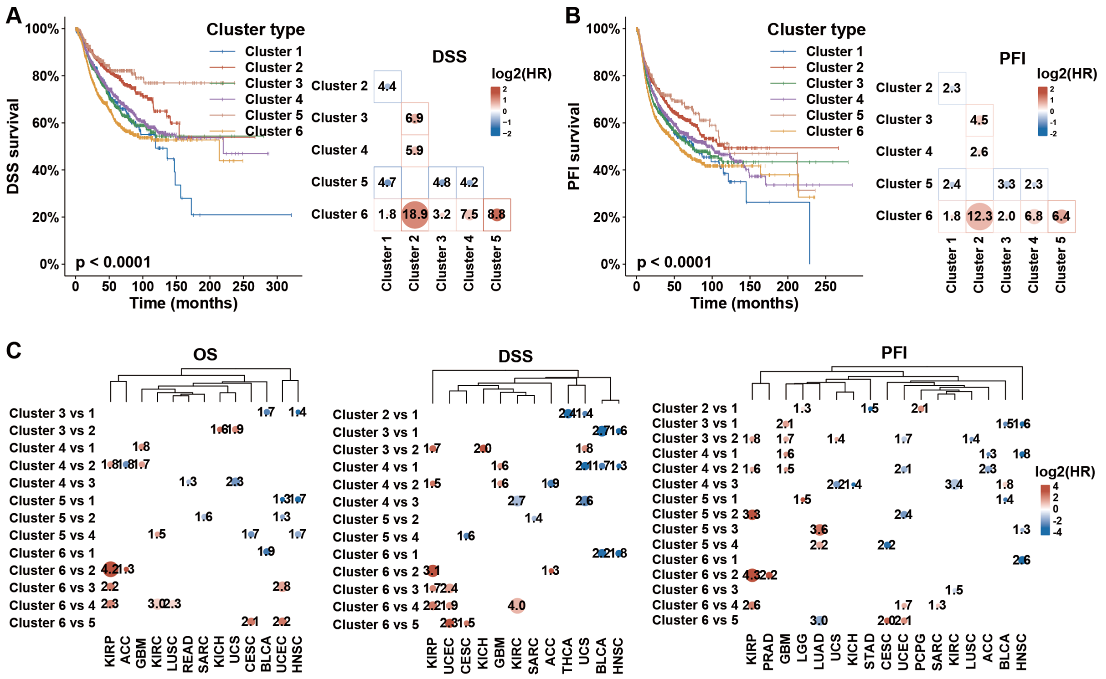

Figure S5. Survival analysis of inflammasome clusters. (A, B) KM curve depicted the disease-specific survival (DSS) and progression-free interval (PFI) of cases from each inflammasome cluster. The p-value was calculate using the log-rank test. Right side plot of the KM curve shows the results of Cox analyses among clusters (clusters in row versus clusters in column). Colors in the plot represented the HR value. The size of the circle and number text represent the negative log10(p-value). Results with p values less than 0.05 are shown. (C) Cox analyses among clusters in each tumor type are performed to evaluate the prognostic value. HR, hazard ratios.

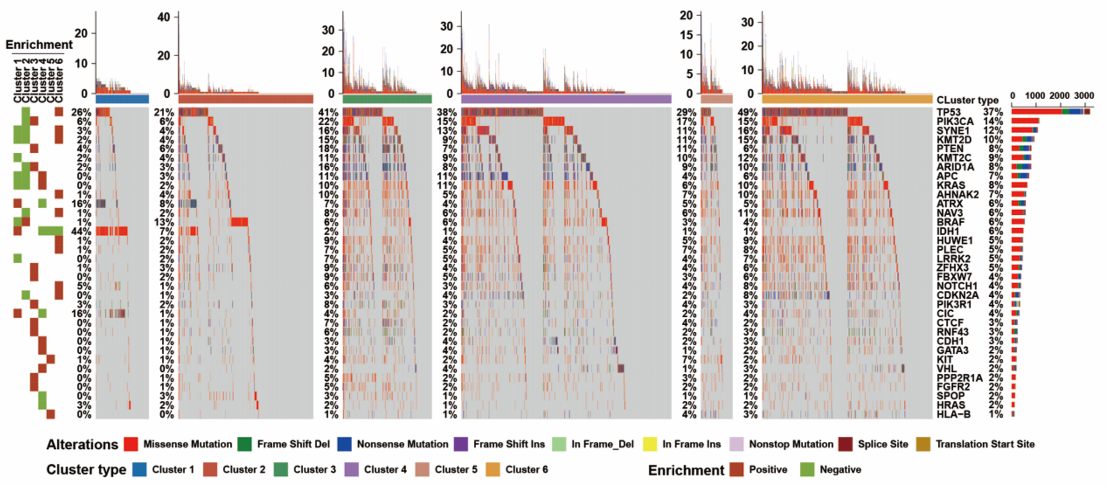

Figure S6. Oncoprint of driver genes differentially mutated in each inflammasome cluster compared to all other clusters. The top 10 significant differentially mutated driver genes assessed using the chi-square test ($p < 0.05$) are depicted. The mutational fraction of each gene in each cluster is shown on the left. The total mutational fraction of each gene across all cases is shown on the right. Row annotation on the left indicate the type of enrichment of the differentially mutated driver genes (significant).

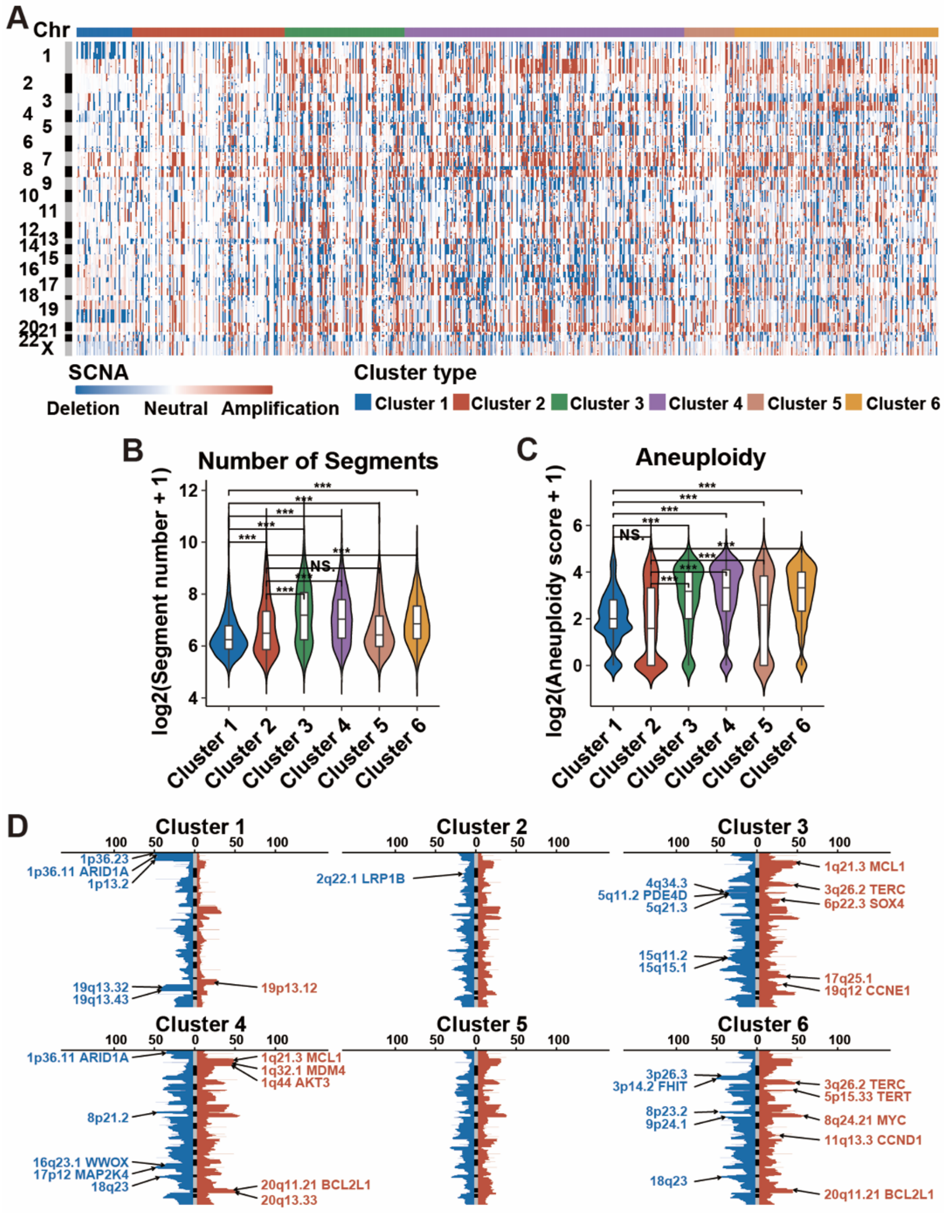

Figure S7. Identification of driver SCNA peaks differentially altered in each inflammasome cluster compared to all other clusters. (A) The overall somatic copy-number alteration (SCNA) profile in clusters. The landscape of amplification (red) and deletion (blue) across the genome is shown in the heatmap. (B) Copy-number segments within each cluster. (C) The distribution of aneuploidy score across clusters. (D) A distinct SCNA profile could be identified among clusters. The horizontal axis indicates the fraction of regions of amplifications (red) and deletions (blue). Text labels indicate the top 5 significant differentially altered driver SCNA peaks identified using the chi-square test ($p < 0.05$), comparing the fraction of driver SCNA peaks in each cluster to that in all other clusters. NS, not significance; ***$p < 0.001$.

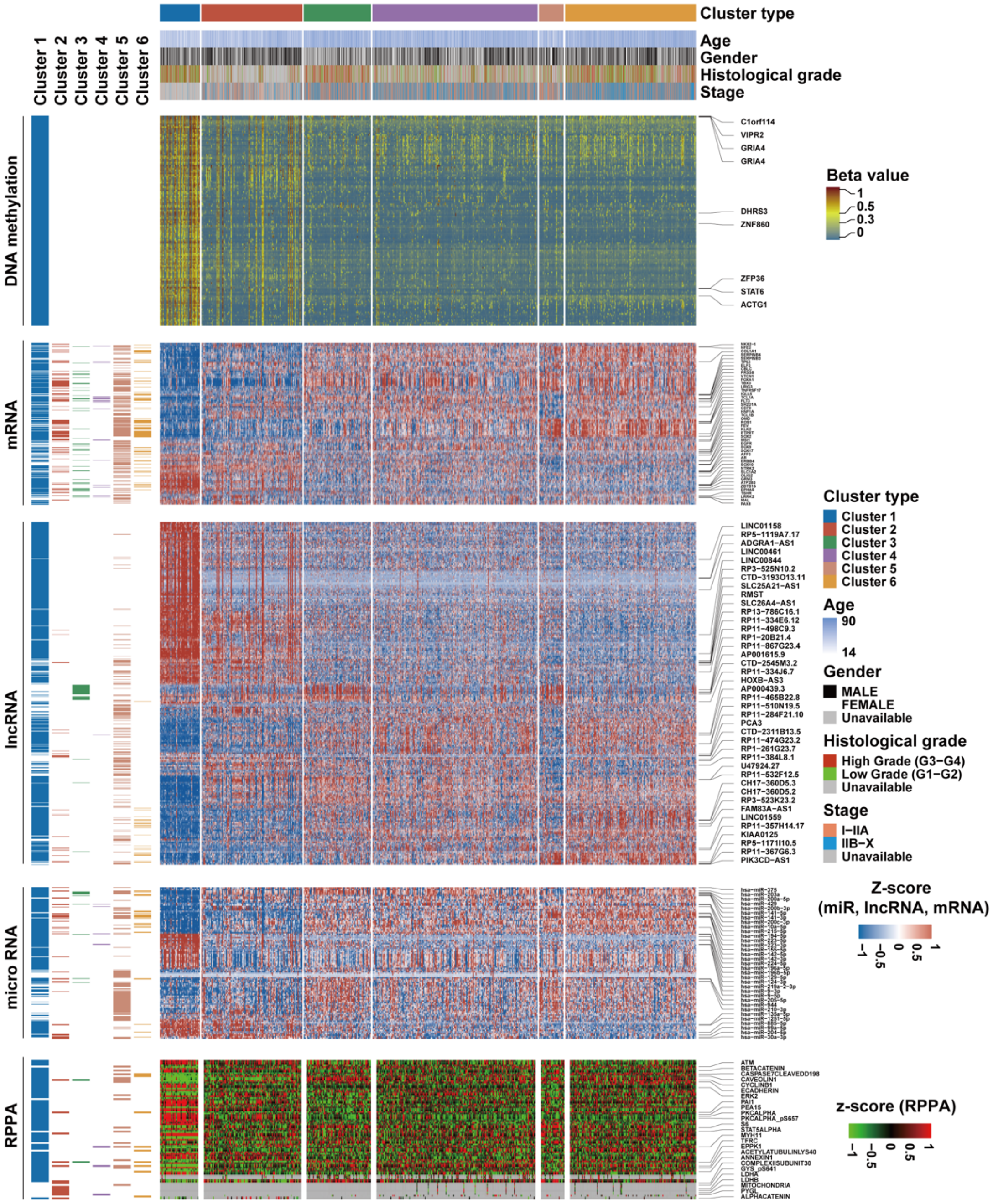

Figure S8. Identification of genomic features differentially altered in each inflammasome cluster compared to all other clusters. Differential methylation level of driver TSS probes between one cluster and all other clusters was evaluated using the champ.DMP function in the ChAMP R package. The differential expression of cancer genes retrieved from the OncoKB website (https://www.oncokb.org/cancerGenes), lncRNAs, and microRNAs was evaluated through the limma R package. The differential protein level between one cluster and all other clusters was evaluated through the Wilcoxon rank-sum test. Only the genomic features that were significantly differentially altered in each inflammasome cluster compared to all other clusters (methylated level: |logFC| > 0.2, FDR < 0.01; cancer genes and miRNA: |logFC| > 1, FDR < 0.01; lncRNA: |logFC| > 2, FDR < 0.01; RPPA: |differential median protein level| > 0.5, FDR < 0.01) are shown in the heatmap. The top 5 up- or down-regulated genomic features in each cluster are labeled on the right side of the heatmap. Row annotation

on the left indicates the enrichment or deletion of the differentially altered genomic features (significant). Grey color in the RPPA panel indicates the unavailable protein level of the related genes in the related samples. RPPA, Reverse Phase Protein Array.

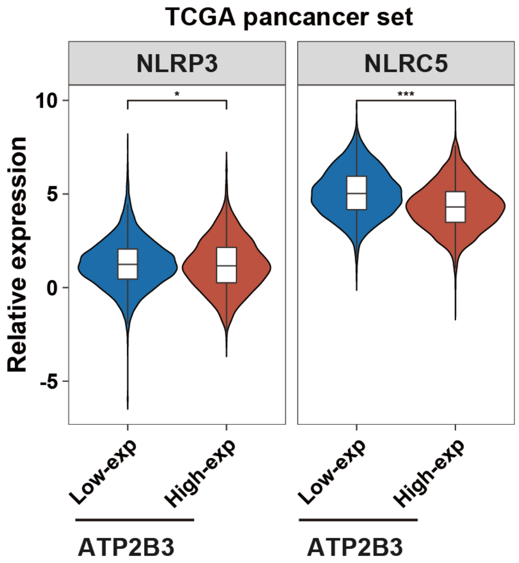

Figure S9. Association between ATP2B3 and NLRP3 and NLRC5. Comparison of NLRP3 and NLRC5 expression between ATP2B3 low- and high-exp groups. The samples with ATP2B3 expression not more than three-quarters of all samples were described as ATP2B3 low expression (Low-exp) group, whereas the other samples as ATP2B3 high expression (High-exp) group. *$p < 0.05$, ***$p < 0.001$.

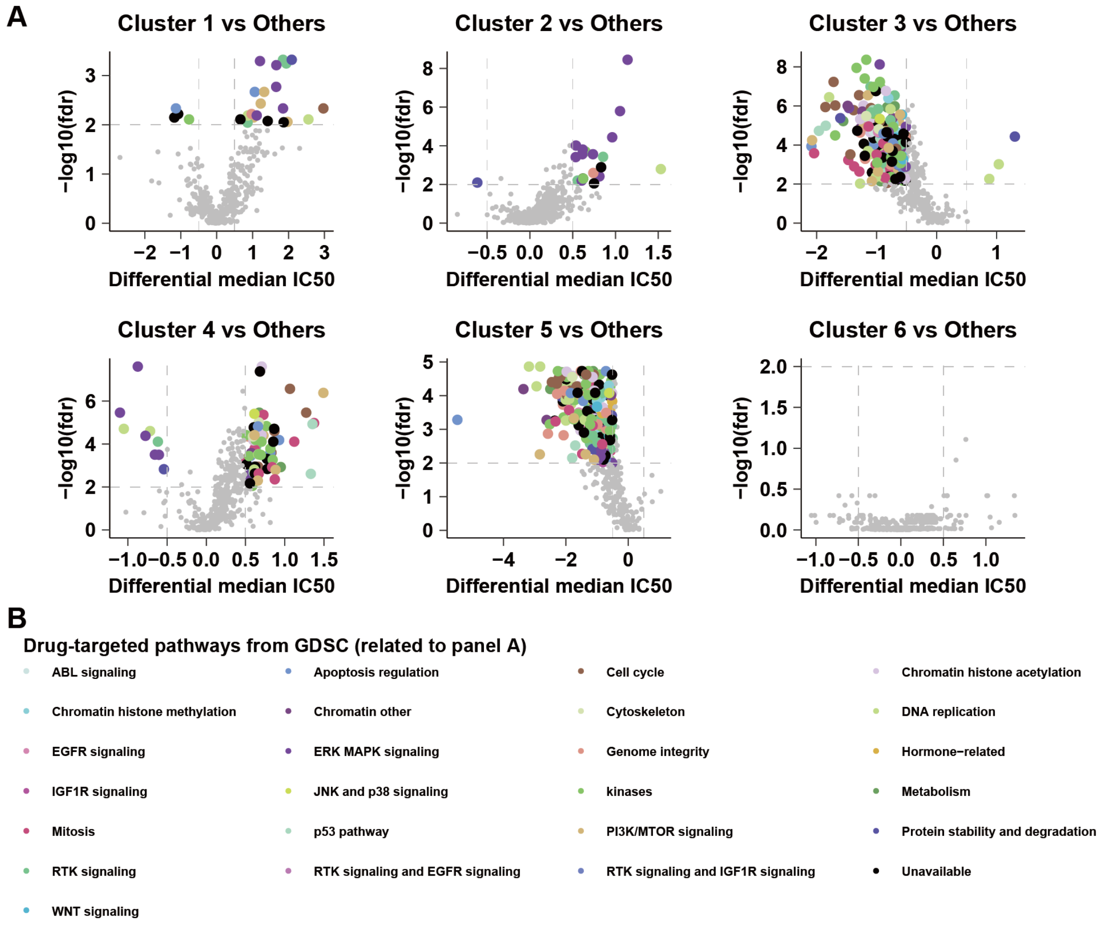

Figure S10. Drug-sensitivity analysis across inflammasome clusters. (A) Differential drug response analysis of cell lines from CCLE with matched drug-sensitive data from Genomics of Drug Sensitivity in Cancer (GDSC) was performed using the Wilcoxon rank-sum test between one cluster and all other clusters (Table S8: Drug sensitivity sheet). Each plot represents a comparison between one cluster and all other clusters, as indicated, and each dot represents one drug. Selected drugs with absolute differential median IC50 more than 0.5 and FDR less than 0.05 are colored according to drug-targeted pathways, as indicated (B). IC50, Half maximal inhibitory concentration.

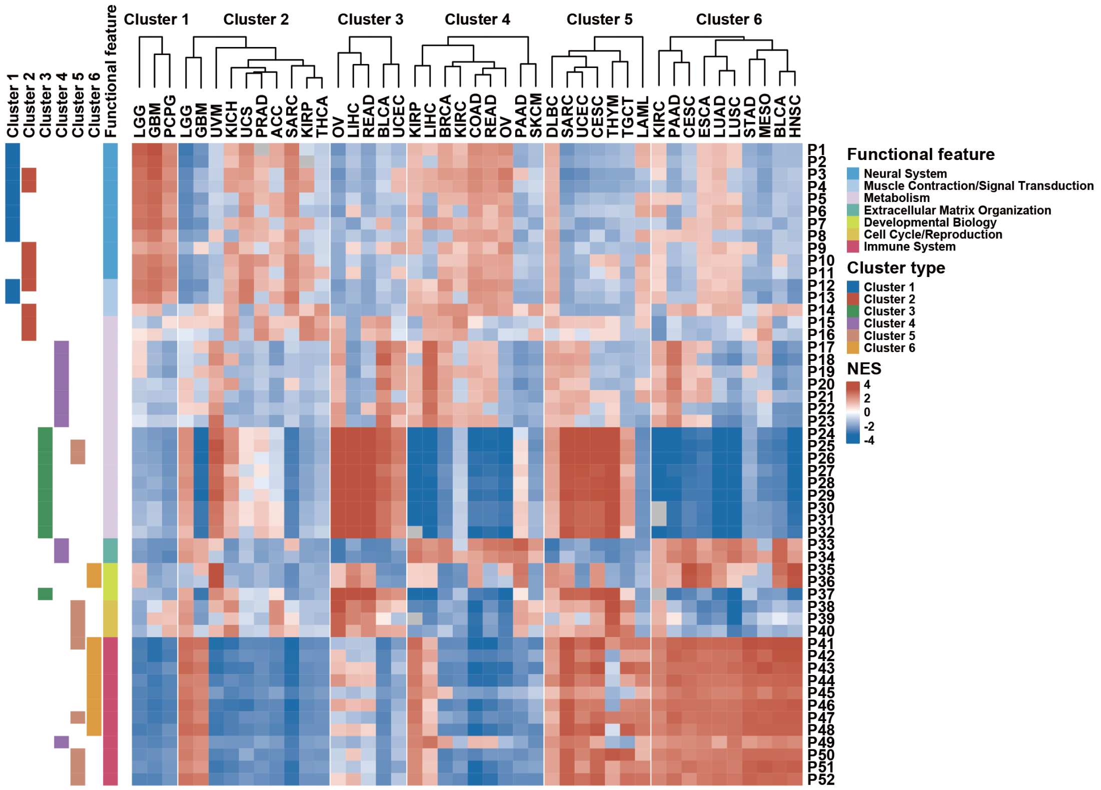

Figure S11. Functional enrichment analysis of each cluster (related to Figure 4A). Functional enrichment analyses of each cluster compared to all other clusters across tumor types were conducted by the GSEA function in the clusterProfiler R package. The pathways with adjusted p < 0.01 and top ten enrichment in each cluster are shown in the heatmap. The NES value is represented by the color intensities. Row annotation on the left indicates the functional features of the related pathways (described in Table S9) and enrichments in related clusters.

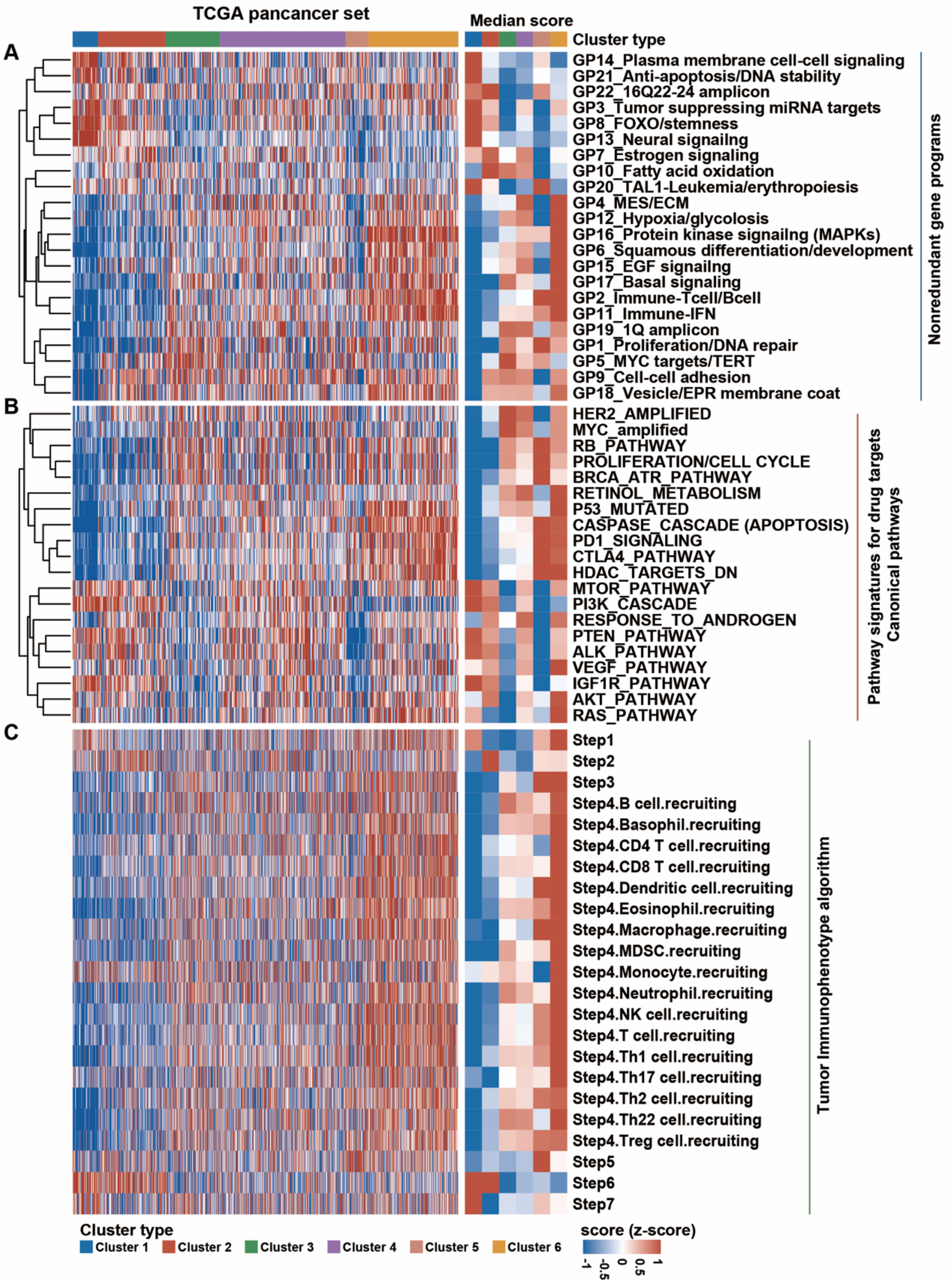

Figure S12. Distinct immune status in different inflammasome clusters. (A and B) The distribution of scores calculated from nonredundant gene programs, pathway signatures for drug targets, and canonical pathways from Hoadley's study across clusters. (C) The distribution of tumor immunophenotype (TIP; http://biocc.hrbmu.edu.cn/TIP/) across clusters.

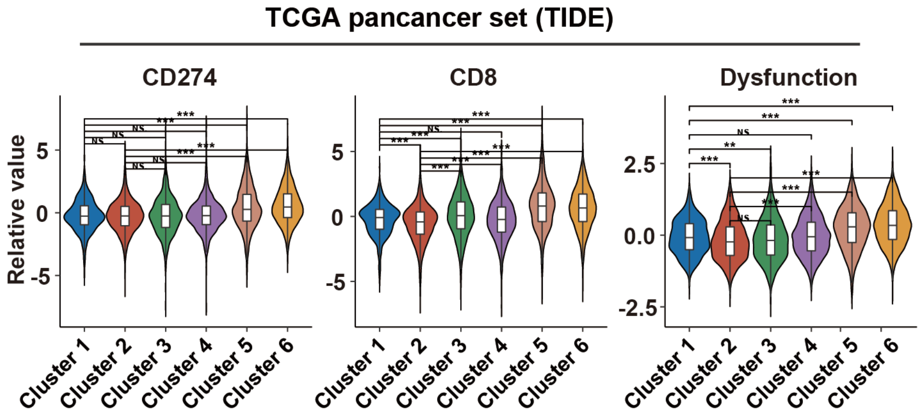

Figure S13. The status of T-cell across clusters was evaluated through TIDE. TIDE, tumor immune dysfunction and exclusion; NS, not significant; **p < 0.01, ***p < 0.001.

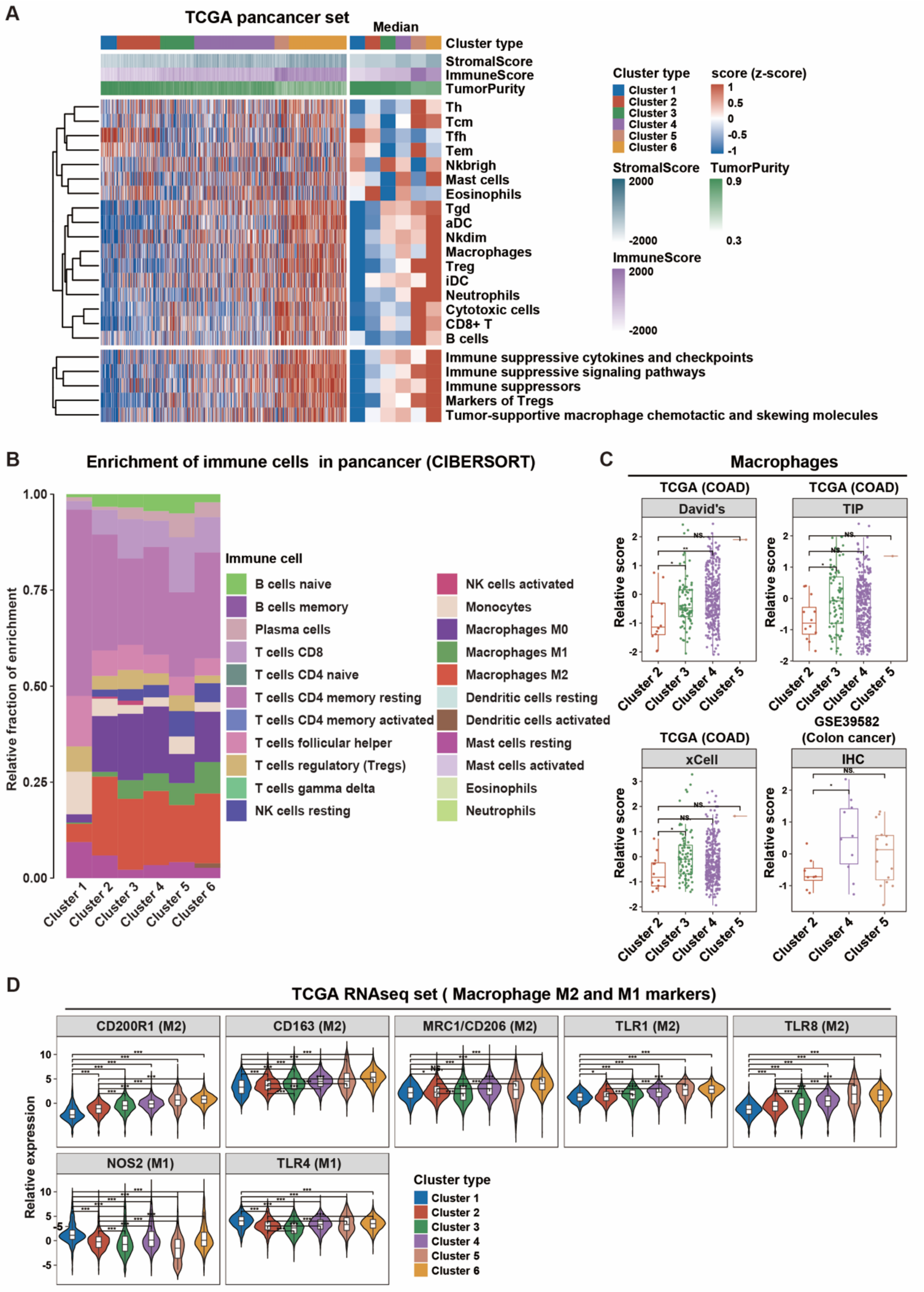

Figure S14. Potential extrinsic mechanism of distinct immune status in different inflammasome clusters. (A) The distribution of the tumor immune microenvironment related scores. The right heatmap indicates the distribution of the median of related scores in each cluster. (B) The distribution of immune cells evaluated by CIBERSORT across clusters. (C) Comparison of macrophage enrichment score among clusters, calculated by ssGSEA from three gene sets in COAD of TCGA and evaluated by the IHC score in colon cancer from GSE39582. (D) Comparison of the expression levels of five M2-related gene

markers [CD200R1, CD163, CD206 (also named MRC1), TLR1, and TLR8] and two M1-related gene markers [NOS2 (also named iNOS) and TLR4] across six inflammasome clusters. IHC, Immunohistochemical; M1, macrophage I phenotype; M2, macrophage II phenotype; NS, not significant; $^{*}p < 0.05$, $^{**}p < 0.01$, $^{***}p < 0.001$.

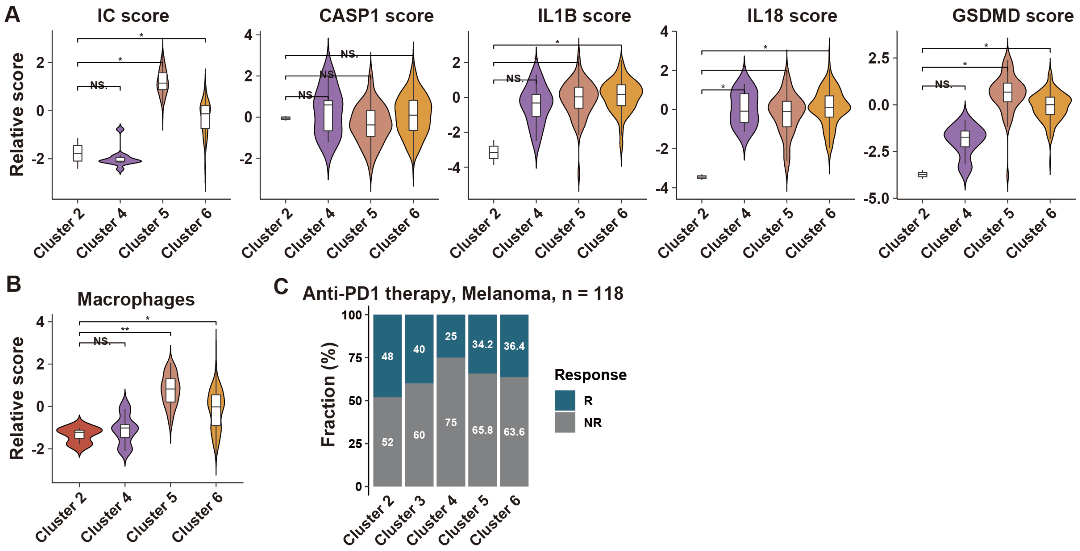

Figure S15. Distribution of inflammasome-signaling-related scores and macrophage enrichment score and ICB therapy response across inflammasome clusters. (A) Comparison of inflammasome-signaling-related scores among clusters in Imvigor210 cohort. (B) Comparison of macrophage enrichment score from David's gene set among clusters in Imvigor210 cohort. (C) The distribution of ICB therapy response across clusters in cutaneous melanoma samples collected prior to anti-PD1 therapy from three cohorts (dbGaP: phs001036, GSE91061, and GSE78220). Only one sample was identified as cluster 1 in these three cohorts (Table S5D), which was not suitable for comparison with other clusters. NS, not significant; *$p < 0.05$, **$p < 0.01$.

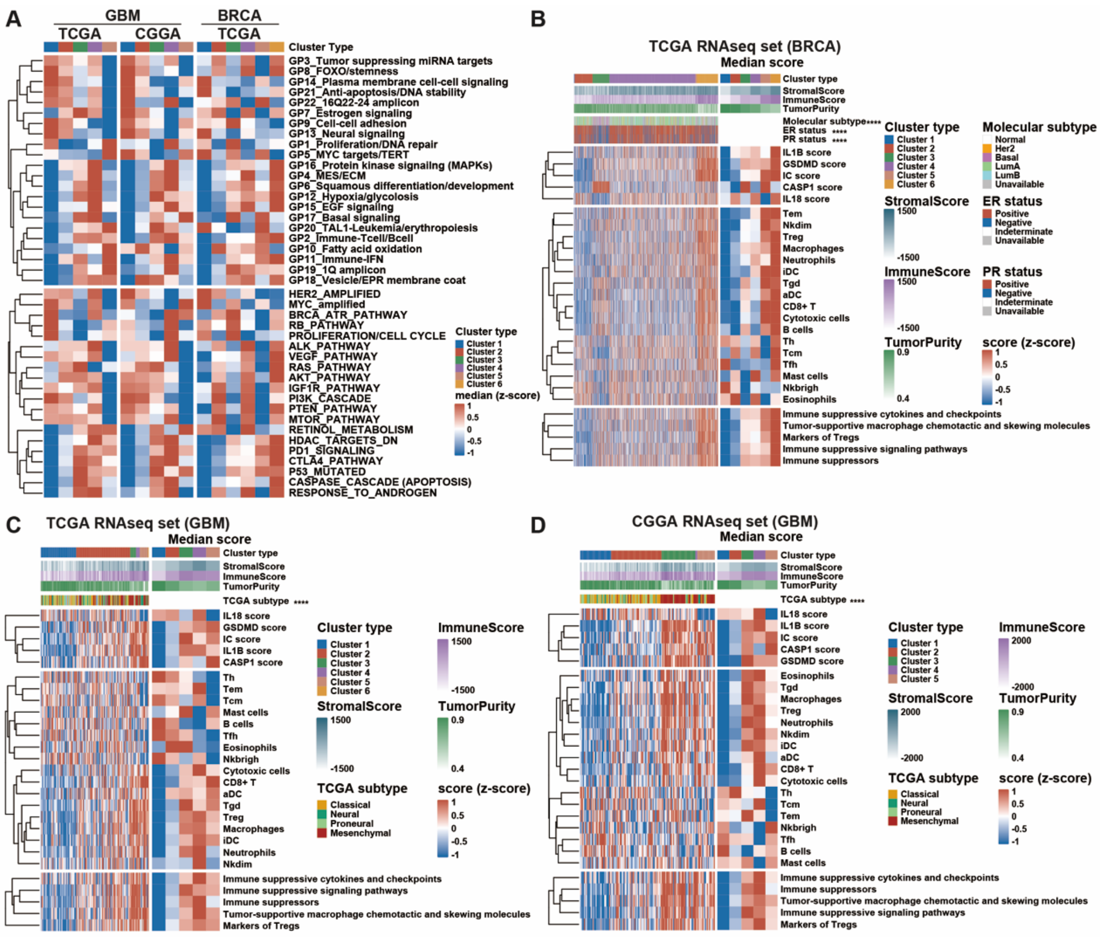

Figure S16. Distinct immune status in different inflammasome clusters of GBM and BRCA. (A) The distribution of median scores calculated from nonredundant gene programs, pathway signatures for drug targets, and canonical pathways from Hoadley's study across various clusters in TCGA-GBM, CGGA-GBM, and TCGA-BRCA. (B-D) The distribution of tumor subtypes, tumor microenvironment scores (stromal score, immune score, and tumor purity), inflammasome-signaling-related scores, enrichment scores of immune cells from David's gene sets, and immunosuppressive gene set scores across various clusters in TCGA-BRCA, TCGA-GBM, and CGGA-GBM. TCGA, The Cancer Genome Atlas; CGGA, the Chinese Glioma Genome Atlas; ****$p < 0.0001$, the p-value was calculated using the chi-square test.

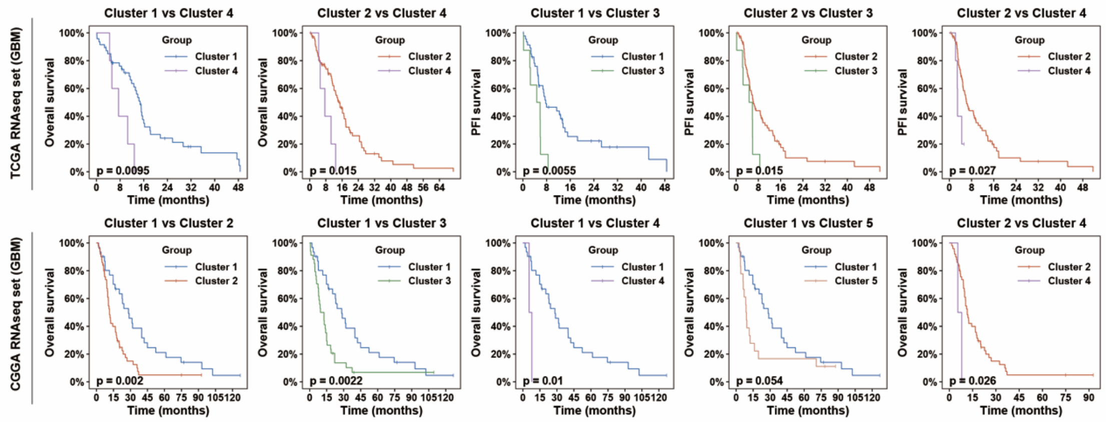

Figure S17. Prognostic value of inflammasome clusters. Kaplan–Meier survival curve was used to evaluate the prognostic value of inflammasome clusters in TCGA-GBM and CGGA-GBM**.**

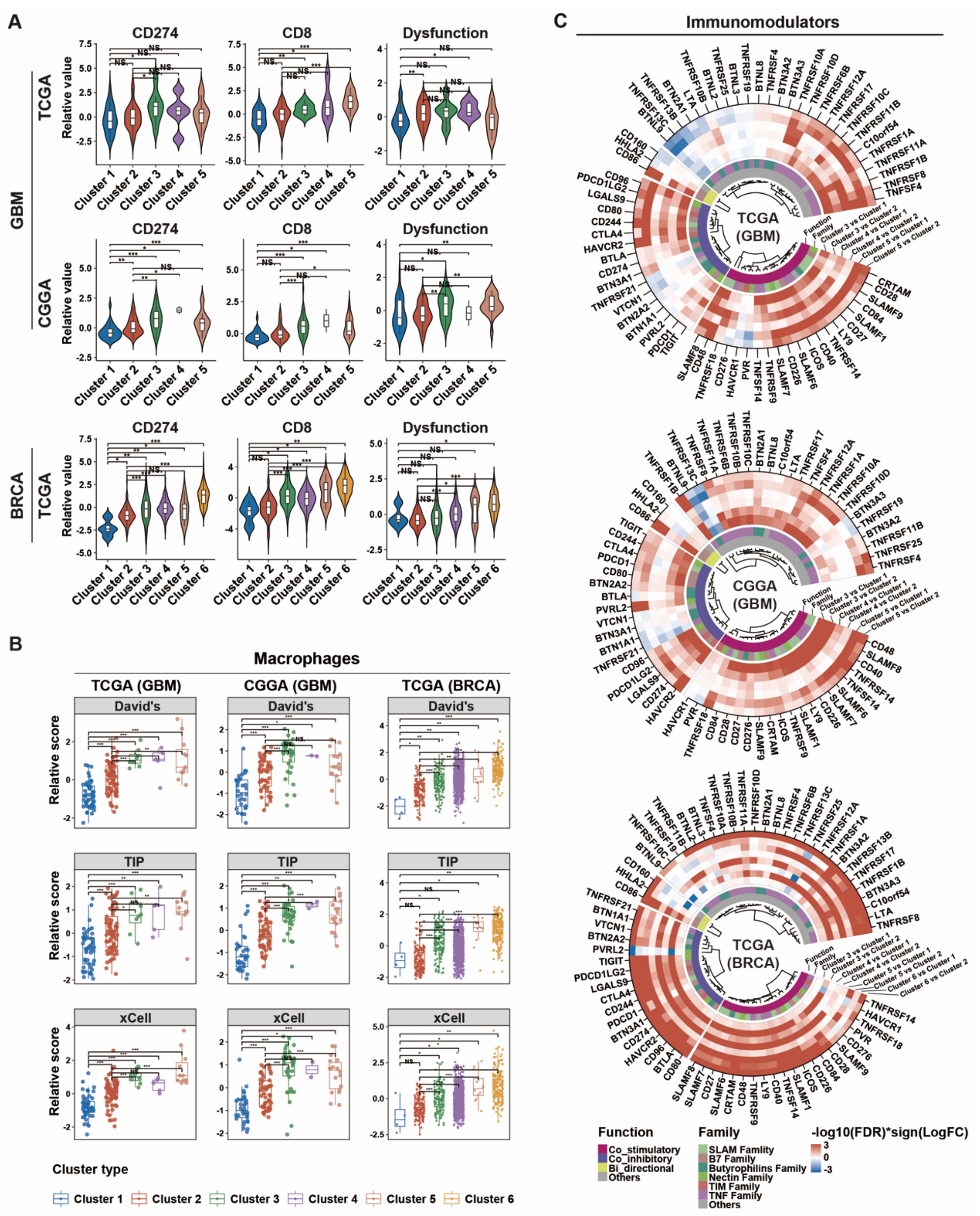

Figure S18. Potential mechanism of distinct immune status in different inflammasome clusters of GBM and BRCA. (A) The status of T-cell across clusters was evaluated through TIDE in TCGA-GBM, CGGA-GBM, and TCGA-BRCA. (B) Comparison of macrophage enrichment score among clusters in TCGA-GBM, CGGA-GBM, and TCGA-BRCA. (C) Immunomodulators profile difference among clusters as indicated was assessed using the Wilcoxon rank-sum test. Color intensities represent negative log10(FDR) multiplied by the sign of the logFC. TIDE, tumor immune dysfunction and exclusion; David's, macrophage related gene set from David's study; TIP, macrophage related gene set from Tumor Immunophenotype (http://biocc.hrbmu.edu.cn/TIP/); xCell, macrophage related gene set from xCell website (https://xcell.ucsf.edu/). NS, not significant; *$p < 0.05$, **$p < 0.01$, ***$p < 0.001$.

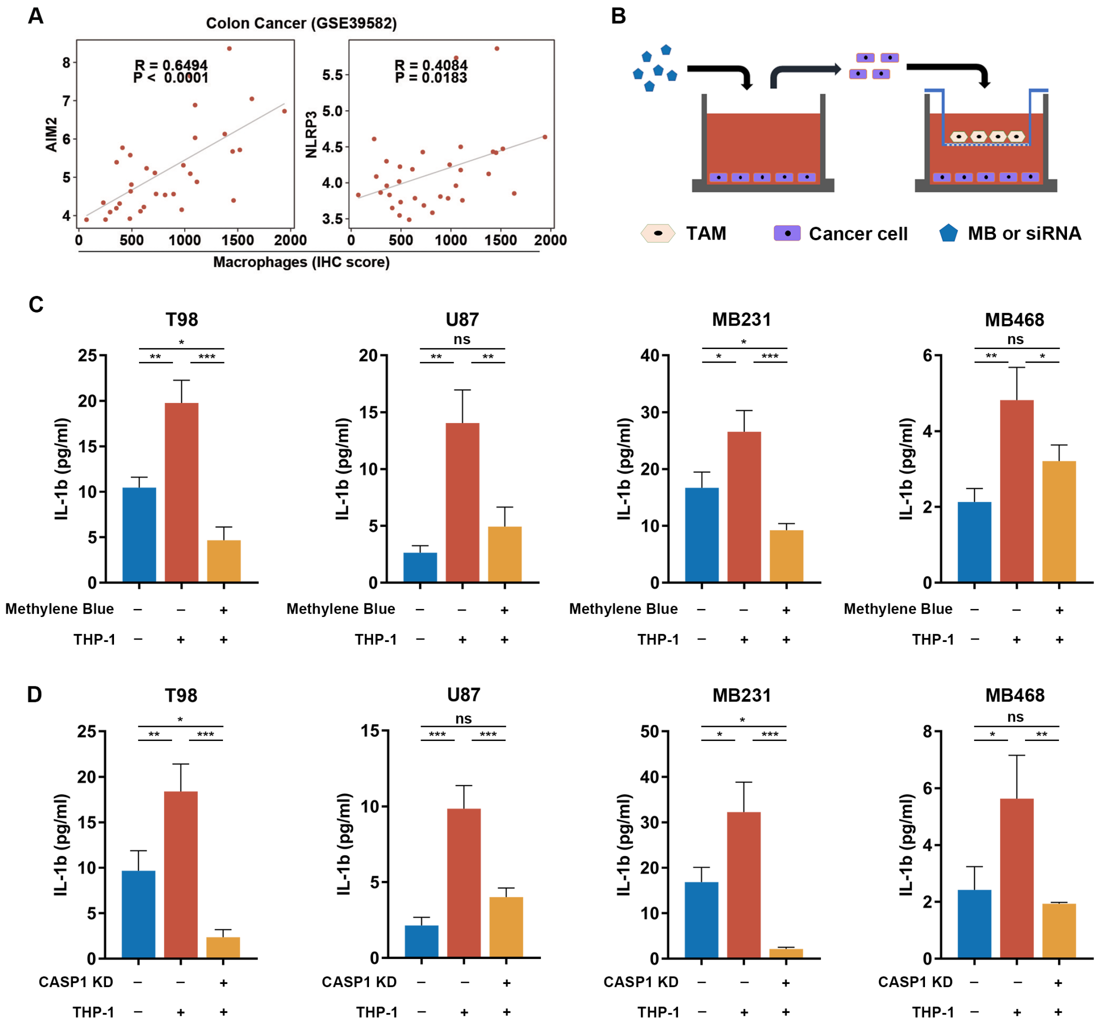

Figure S19. Methylene blue and CASP1 knockdown abrogate TAM-mediated inflammasome activation in cancer cells. (A) Pearson correlation analysis of the expression level of IRGs (AIM2 and NLRP3) and macrophage enrichment score represented using IHC score in colon cancer from GSE39582. (B) Experiment procedure of cancer cells and TAM co-culturing. (C and D) ELISA analysis of the concentration of IL-1β in 24 h conditioned medium of TAM co-cultured cancer cells that were pre-treated with MB (C) or CASP1 targeted siRNA (D). ns, not significant; *p < 0.05, **p < 0.01, ***p < 0.001.

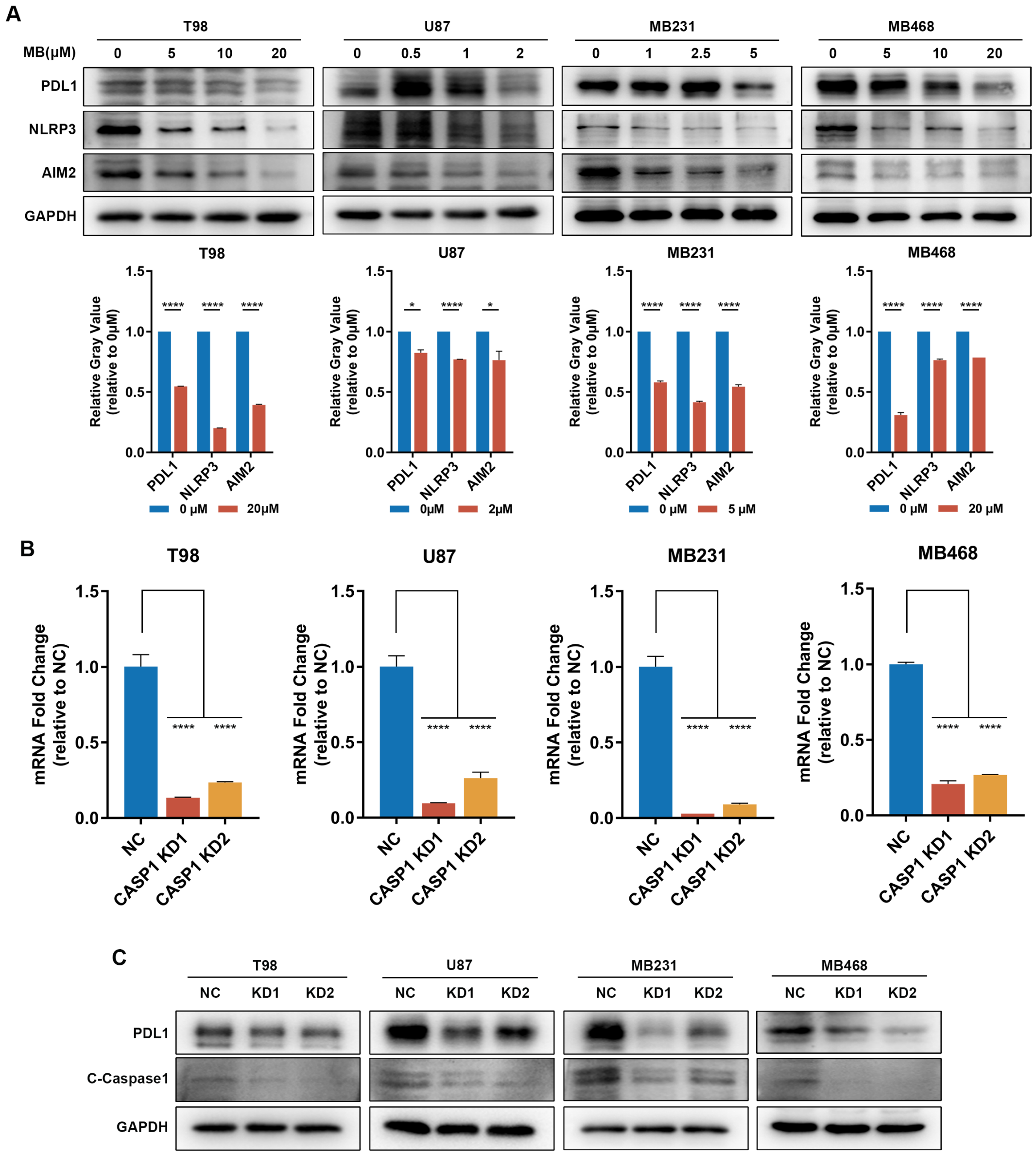

Figure S20. Methylene blue and CASP1 knockdown inhibit inflammasome signaling activation and PD-L1 expression in cancer cells. (A) Western blotting analysis and quantification of inflammasomes (NLRP3 and AIM) and PD-L1 expression in GBM and BRCA cell lines treated with indicated doses of MB for 48 h. (B–C) Real-time PCR analysis (B) and western blotting analysis (C) of the CASP1 knockdown efficacy and PD-L1 expression in cancer cells treated with CASP1 targeted siRNAs. *$p < 0.05$, ****$p < 0.0001$.

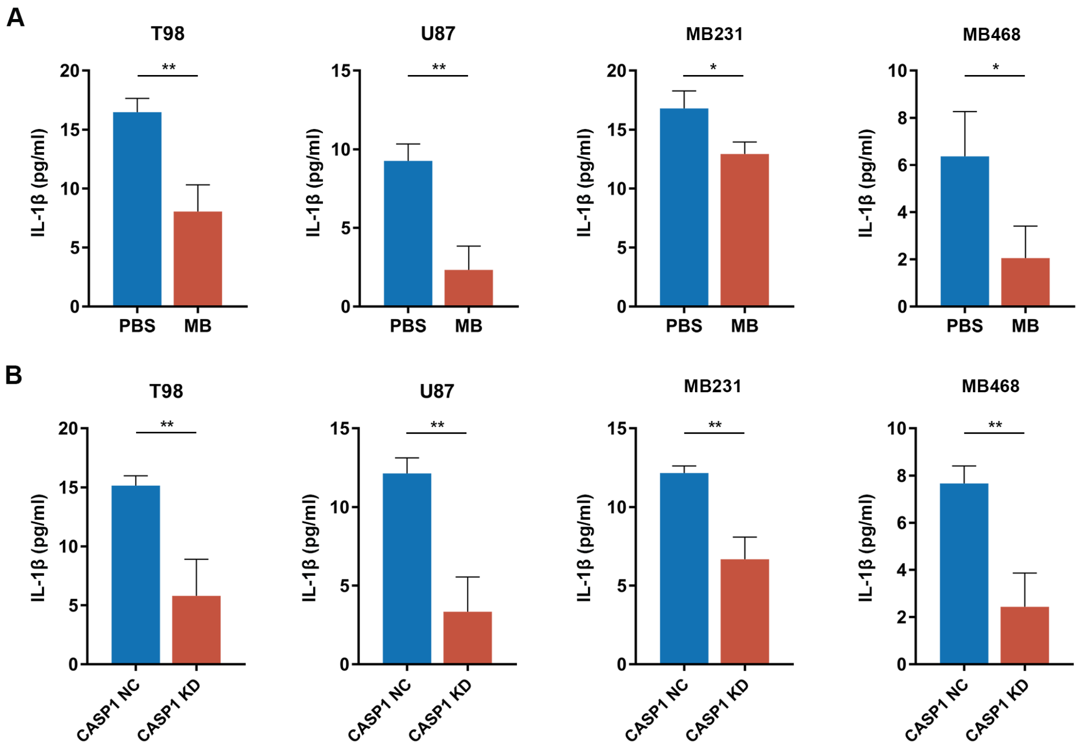

Figure S21. Inflammasome signaling inhibition effectively downregulates IL-1β expression in cancer cells. (A–B) ELISA analysis of the concentration of IL-1β in 24 h conditioned medium of cancer cells treated with MB (A) or CASP1 targeted siRNAs (B). $*p < 0.05$, $**p < 0.01$.

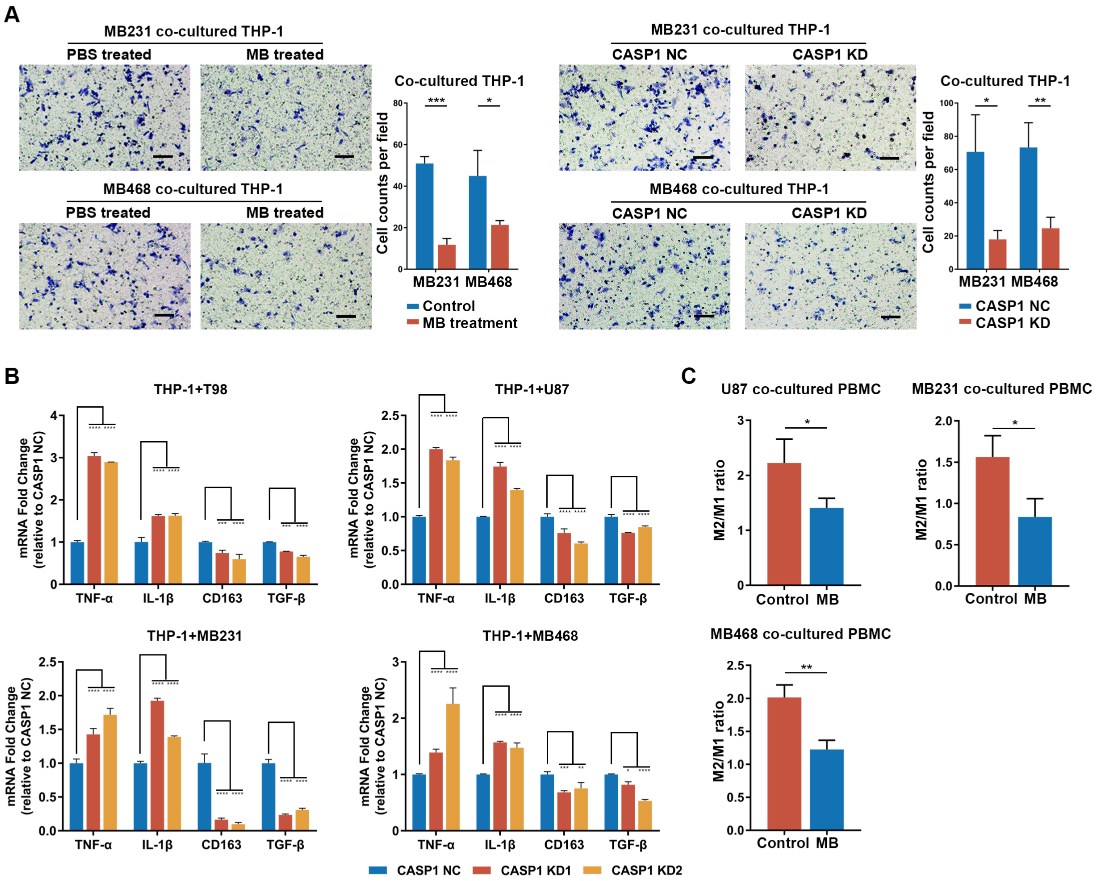

Figure S22. Inflammasome inhibition in cancer cells reduces immunosuppressive features of TAM. (A) Migration analysis of THP-1 co-cultured with cancer cells pre-treated with MB or CASP1 targeted siRNA. Scale bar: 25 μm. (B) Real-time PCR analysis of the M1 (TNF-α, IL-1β)/M2 (CD163, TGF-β) markers in THP-1 which was co-cultured with CASP1 targeted siRNAs pre-treated cancer cells. (C) Flow cytometry analysis of the M2 ($CD206^{+}$) and M1($MHCII^{+}$) polarization tendency of PBMC (Peripheral Blood Mononuclear Cell). *$p < 0.05$, **$p < 0.01$, ***$p < 0.001$, ****$p < 0.0001$.

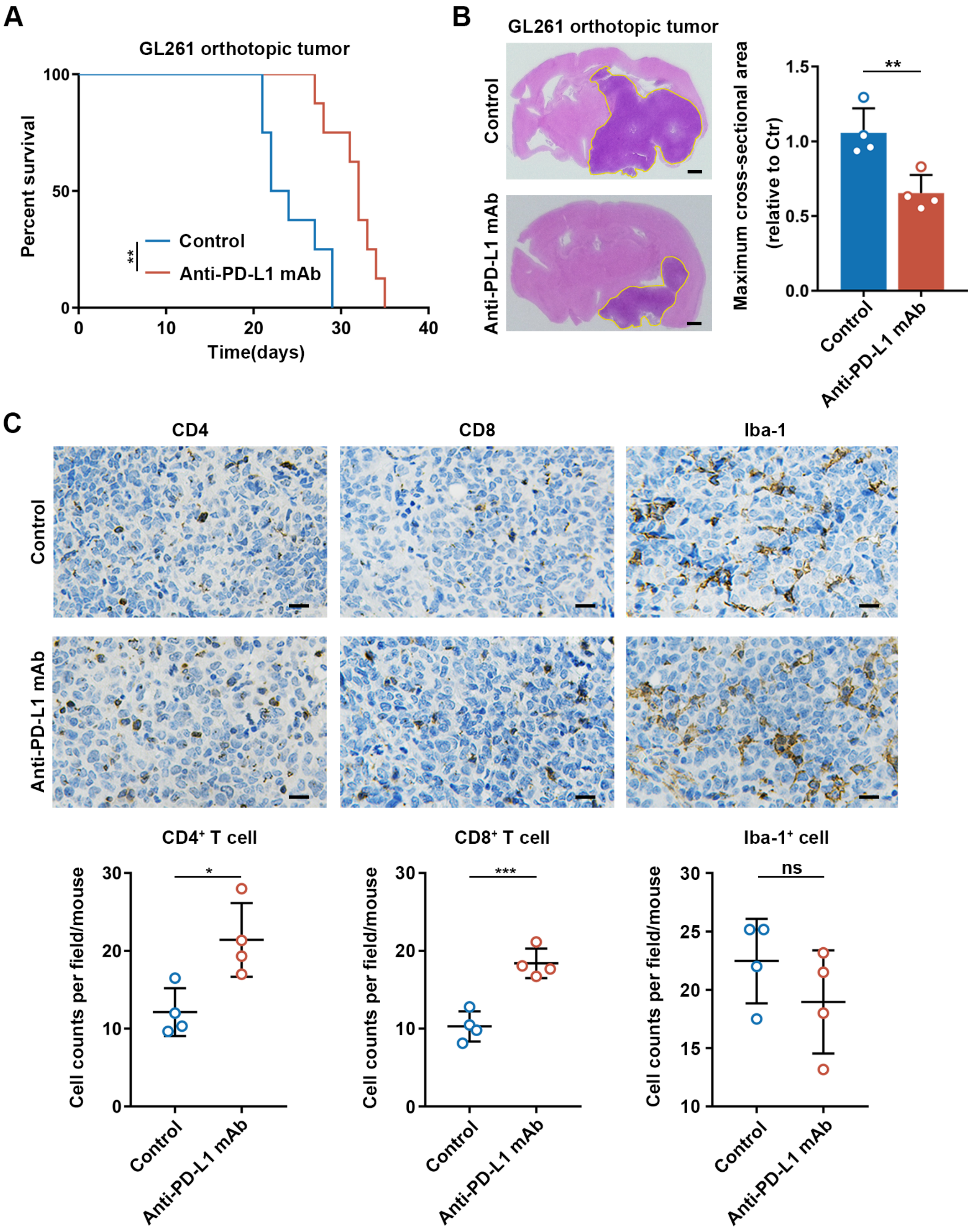

Figure S23. GL261 GBM model is sensitive to PD-L1 blockade therapy. (A) Survival analysis of GL261 model treated with PD-L1 neutralizing antibody. (B) Hematoxylin and eosin staining and quantification of the relative maximum cross-sectional area of orthotopic tumors treated with PD-L1 antibody. Scale bar: 500 µm. (C) Immunohistochemistry analysis and quantitative cell counts of infiltrated immune cells in orthotopic tumors. Scale bar: 25 µm. *$p < 0.05$, **$p < 0.01$, ***$p < 0.001$.

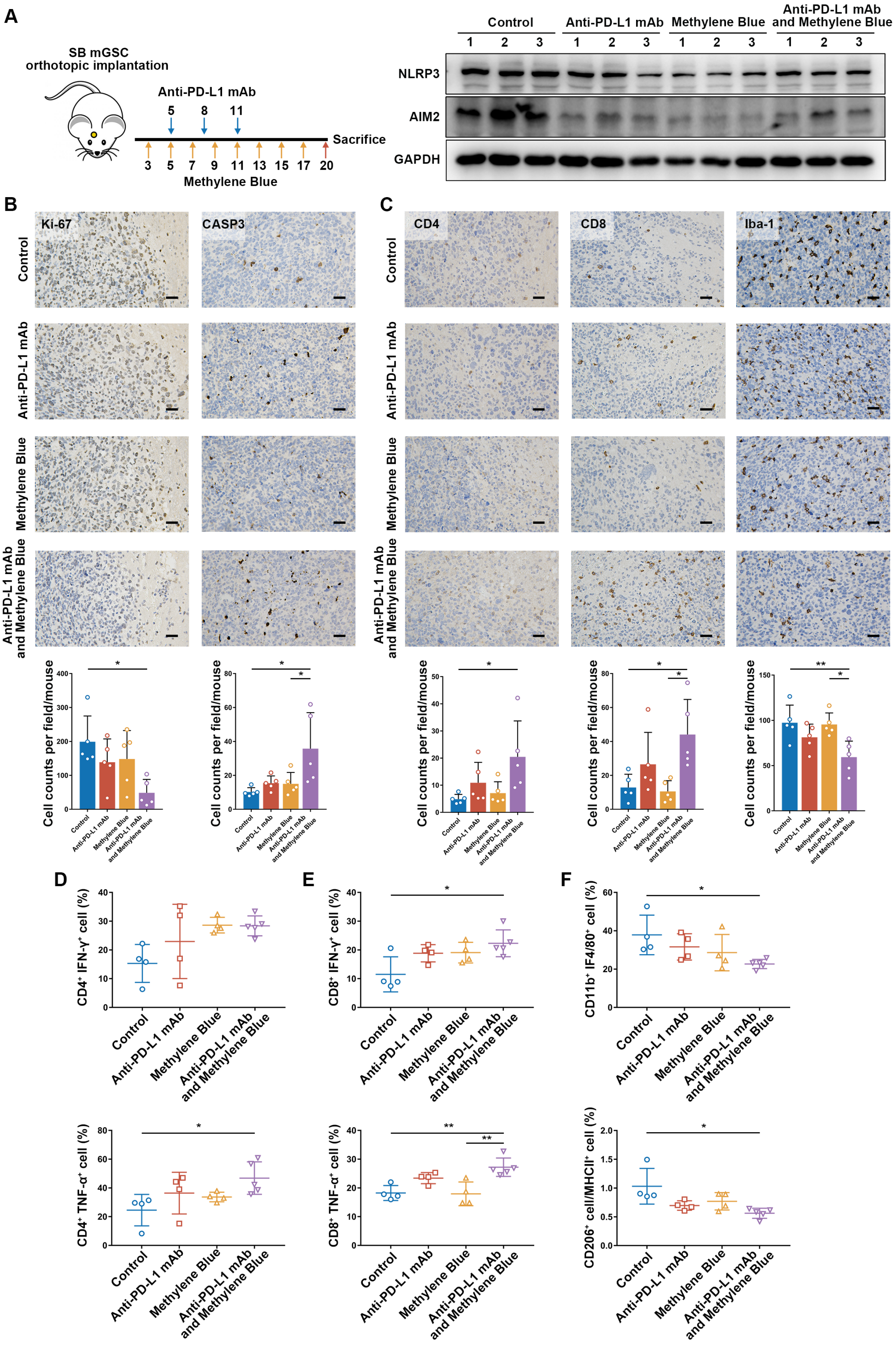
A
SB mGSC
orthotopic implantation
Anti-PD-L1 mAb
5
8
11
Sacrifice
3
5
7
9
11
13
15
17
20
Methylene Blue
Control
Anti-PD-L1 mAb
Methylene Blue
Anti-PD-L1 mAb
and Methylene Blue
1
2
3
NLRP3
AIM2
GAPDH
B
C
Ki-67
CASP3
CD4
CD8
Iba-1
Control
Anti-PD-L1 mAb
Methylene Blue
Anti-PD-L1 mAb
and Methylene Blue
Cell counts per field/mouse
D
E
F
CD4+ IFN-γ+ cell (%)
CD8+ IFN-γ+ cell (%)
CD11b+ IF4/80+ cell (%)
CD4+ TNF-α+ cell (%)
CD8+ TNF-α+ cell (%)
CD206+ cell/MHCII+ cell (%)

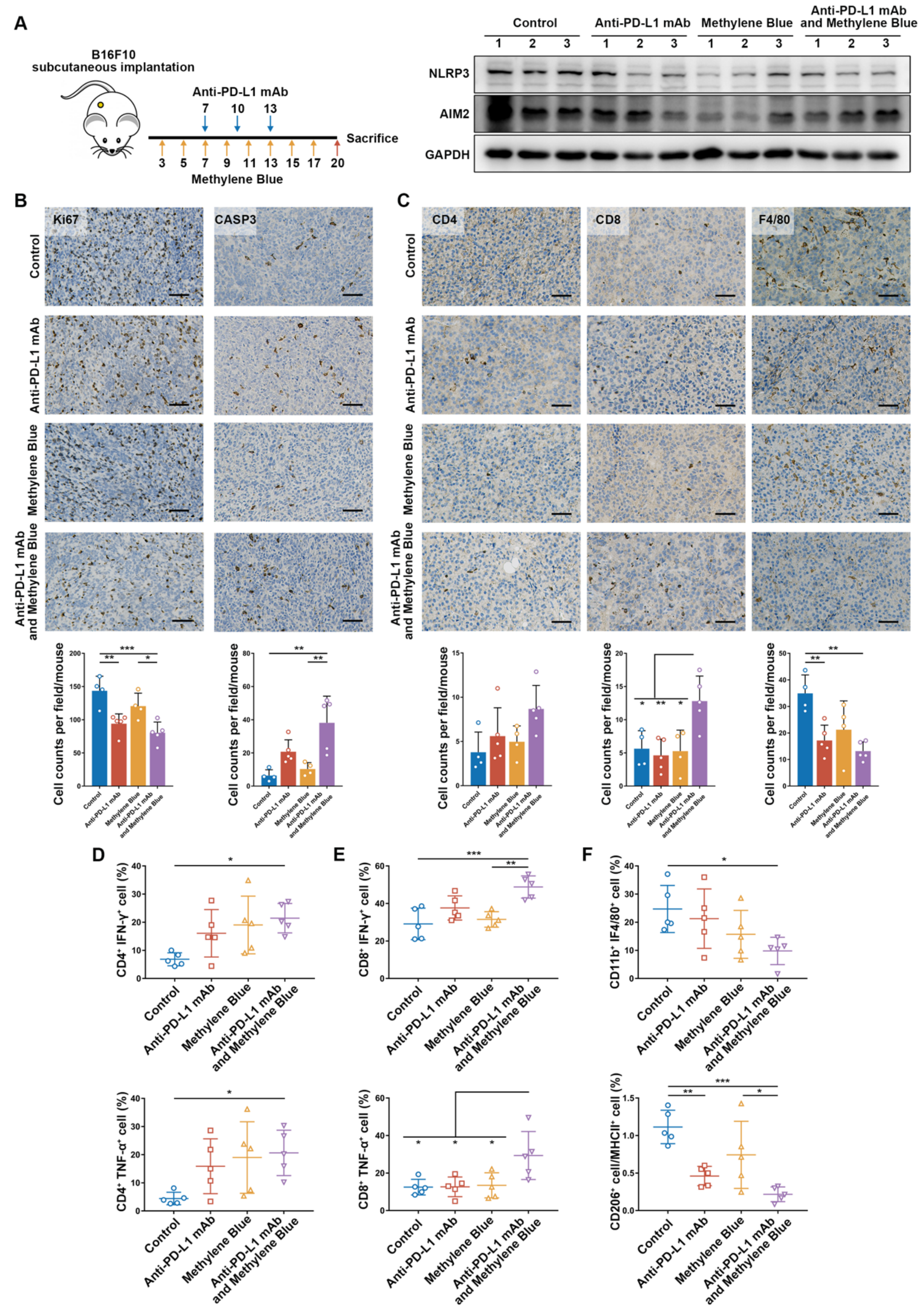

Figure S24 and S25. Methylene blue treatment combined with PD-L1 blockade suppresses tumor progression by improving anti-tumor immune environment in SB mGSC GBM model (Figure S24) and B16F10 melanoma model (Figure S25). (A) Drug administration schedule and western blotting analysis of the inflammasome inhibition efficacy of MB in tumors. (B) Immunohistochemistry analysis of Ki-67 and cleaved-caspase 3 expressions in tumors. Scale bar: 50 μm. (C) Immunohistochemistry analysis and quantitative cell counts of infiltrated immune cells in orthotopic tumors. Scale bar: 50 μm.

(D to F) Flow cytometry analysis of the proportion of infiltrated $CD4^+$ T cell populations (D), $CD8^+$ T cell populations (E), and TAM (F) in tumors. *p < 0.05, **p < 0.01, ***p < 0.001.

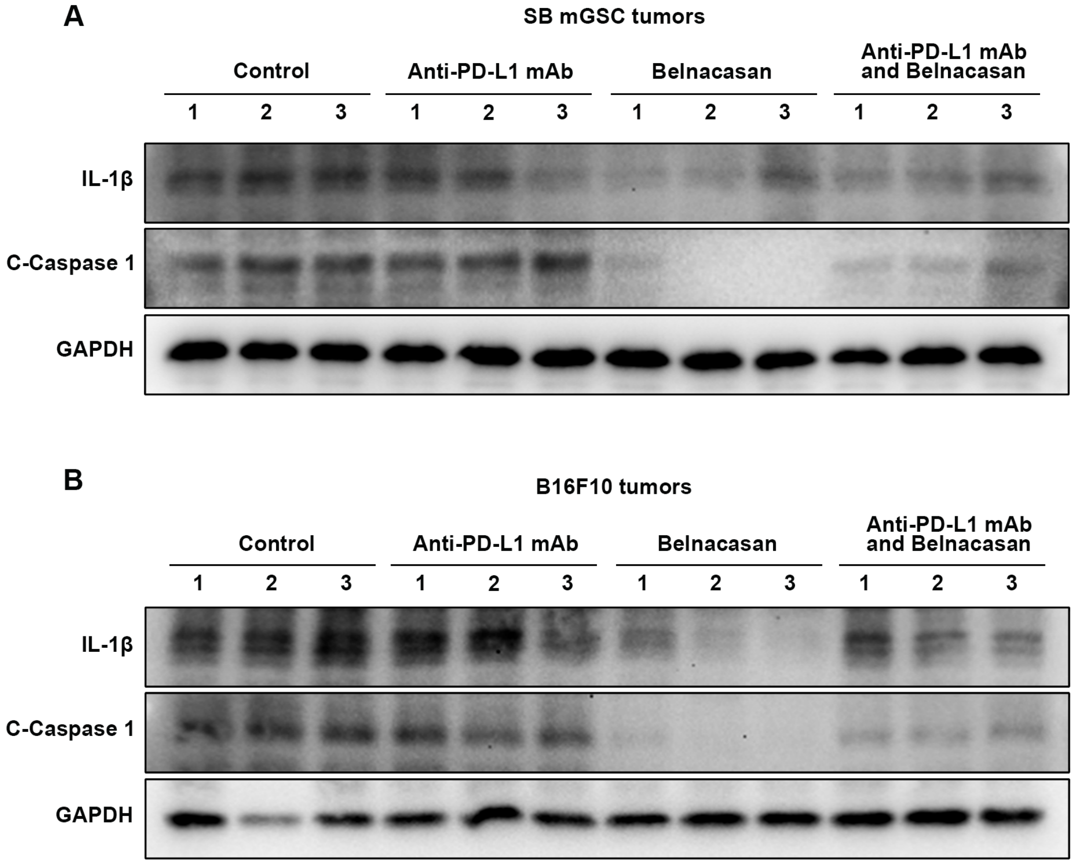

Figure S26. Belnacasan treatment combined with PD-L1 blockade suppresses inflammasome signaling activation in tumors. (A and B) Western blotting analysis of the cleaved-caspase 1 inhibition efficacy of Belnacasan (IL-1β and cleaved-caspase 1 expression) in tumors.
